# Tetraspanins distinguish separate extracellular vesicle subpopulations in human serum and plasma – Contributions of platelet extracellular vesicles in plasma samples

**DOI:** 10.1002/jev2.12213

**Published:** 2022-05-06

**Authors:** Nasibeh Karimi, Razieh Dalirfardouei, Tomás Dias, Jan Lötvall, Cecilia Lässer

**Affiliations:** ^1^ Krefting Research Centre Department of Internal Medicine and Clinical Nutrition Institute of Medicine Sahlgrenska Academy University of Gothenburg Gothenburg Sweden; ^2^ Endometrium and Endometriosis Research Center Hamadan University of Medical Sciences Hamadan Iran; ^3^ Faculty of Medicine Department of Medical Biotechnology Mashhad University of Medical Sciences Mashhad Iran; ^4^ Mursla Ltd. Cambridge UK

**Keywords:** biomarkers, exosomes, extracellular vesicles, microvesicles, plasma, serum, subpopulations

## Abstract

**Background**: The ability to isolate extracellular vesicles (EVs) from blood is vital in the development of EVs as disease biomarkers. Both serum and plasma can be used, but few studies have compared these sources in terms of the type of EVs that are obtained. The aim of this study was to determine the presence of different subpopulations of EVs in plasma and serum.

**Method**: Blood was collected from healthy subjects, and plasma and serum were isolated in parallel. ACD or EDTA tubes were used for the collection of plasma, while serum was obtained in clot activator tubes. EVs were isolated utilising a combination of density cushion and SEC, a combination of density cushion and gradient or by a bead antibody capturing system (anti‐CD63, anti‐CD9 and anti‐CD81 beads). The subpopulations of EVs were analysed by NTA, Western blot, SP‐IRIS, conventional and nano flow cytometry, magnetic bead ELISA and mass spectrometry. Additionally, different isolation protocols for plasma were compared to determine the contribution of residual platelets in the analysis.

**Results**: This study shows that a higher number of CD9^+^ EVs were present in EDTA‐plasma compared to ACD‐plasma and to serum, and the presence of CD41a on these EVs suggests that they were released from platelets. Furthermore, only a very small number of EVs in blood were double‐positive for CD63 and CD81. The CD63^+^ EVs were enriched in serum, while CD81^+^ vesicles were the rarest subpopulation in both plasma and serum. Additionally, EDTA‐plasma contained more residual platelets than ACD‐plasma and serum, and two centrifugation steps were crucial to reduce the number of platelets in plasma prior to EV isolation.

**Conclusion**: These results show that human blood contains multiple subpopulations of EVs that carry different tetraspanins. Blood sampling methods, including the use of anti‐coagulants and choice of centrifugation protocols, can affect EV analyses and should always be reported in detail.

## 1 INTRODUCTION

Extracellular vesicles (EVs) are lipid bilayer‐delimited nano‐sized structures that can communicate biological signals between cells, thus regulating activities of recipient cells through the transfer of functional bioactive molecules from the producing cell. During their biogenesis, EVs are loaded with cargo molecules, including lipids, RNA species and cytoplasmic proteins as well as surface receptors associated with the EV membrane.

EVs are a promising new class of biomarkers because of their presence in several biofluids such as blood, urine and saliva and because of their ability to protect intravesicular molecules such as RNA (Keller et al., 2011; Lässer et al., 2011). Importantly, EV cargo can represent the status of its producing cell and can contain, for example, cancer‐associated molecules (Lässer, 2015). These findings have raised the interest in utilising EVs as diagnostic, prognostic and treatment‐response biomarkers for several different cancers and inflammatory diseases. For example, EV‐associated RNAs and proteins have been suggested as biomarkers for glioblastoma, malignant melanoma, prostate cancer and asthma (Dhondt et al., 2020; Jang et al., 2019; Levanen et al., 2013; Skog et al., 2008).

The ability to purify or enrich EVs from blood is vital for biomarker discovery studies. Blood is one of the most routinely collected biofluids, but it is the most complex biofluid to analyse (Ruhen & Meehan, 2019; Simonsen, 2017; Zhou et al., 2020). Some of the challenges associated with studying EVs in plasma and serum include contamination from soluble proteins such as albumin and fibrinogen and co‐isolation of lipoprotein particles with the EVs (Simonsen, 2017). With this knowledge, multiple techniques have been used to deplete the most abundant plasma proteins and lipoprotein particles from circulating EV isolates (Boing et al., 2014; Tulkens et al., 2020; Van Deun et al., 2020; Welton et al., 2016), with varied success. We previously developed a novel method to isolate EVs from plasma by applying the combination of density cushion and size‐exclusion chromatography (SEC), resulting in minimal contamination of lipoprotein particles in the isolated EVs (Karimi et al., 2018).

Both serum and plasma contain EVs and have been used for EV biomarker discovery, although plasma is more commonly used (Gardiner et al., 2016). Plasma and serum are similar in composition; however, serum contains fewer cells and has a smaller amount of soluble proteins such as fibrinogen due to removal of the fibrin clot. There has been some discussion in the EV field as to whether plasma or serum should be recommended for EV biomarker studies. Most often plasma has been suggested to be preferred over serum due to the concern that the ex vivo clotting process could lead to the production of EVs specifically from activated platelets, or that it could lead to the consumption of EVs that are present in the circulation. However, few studies have systematically compared plasma and serum EVs side by side in terms of the actual numbers and phenotypes of EVs that are present and that can be isolated. The aim of this study was therefore to compare the phenotypes of different subpopulations of EVs present in plasma and serum. For this purpose, we isolated EVs from paired serum and plasma samples collected from healthy volunteers using three different EV isolation procedures.

## 2 METHODS

The following tubes were used in the present study: 15‐ml Screw Cap Tube Conical Bottom, (catalogue number 62.554.502, Sarstedt, Numbrecht, Germany), 50‐ml Screw Cap Tube Conical Bottom, (catalogue number 62.547.254, Sarstedt) and 1.5‐ml Microcentrifuge Tubes, (catalogue number FB74031, Fisher Scientific, Waltham, MA, USA).

### 2.1 Plasma and serum preparation

**FIGURE 1:**
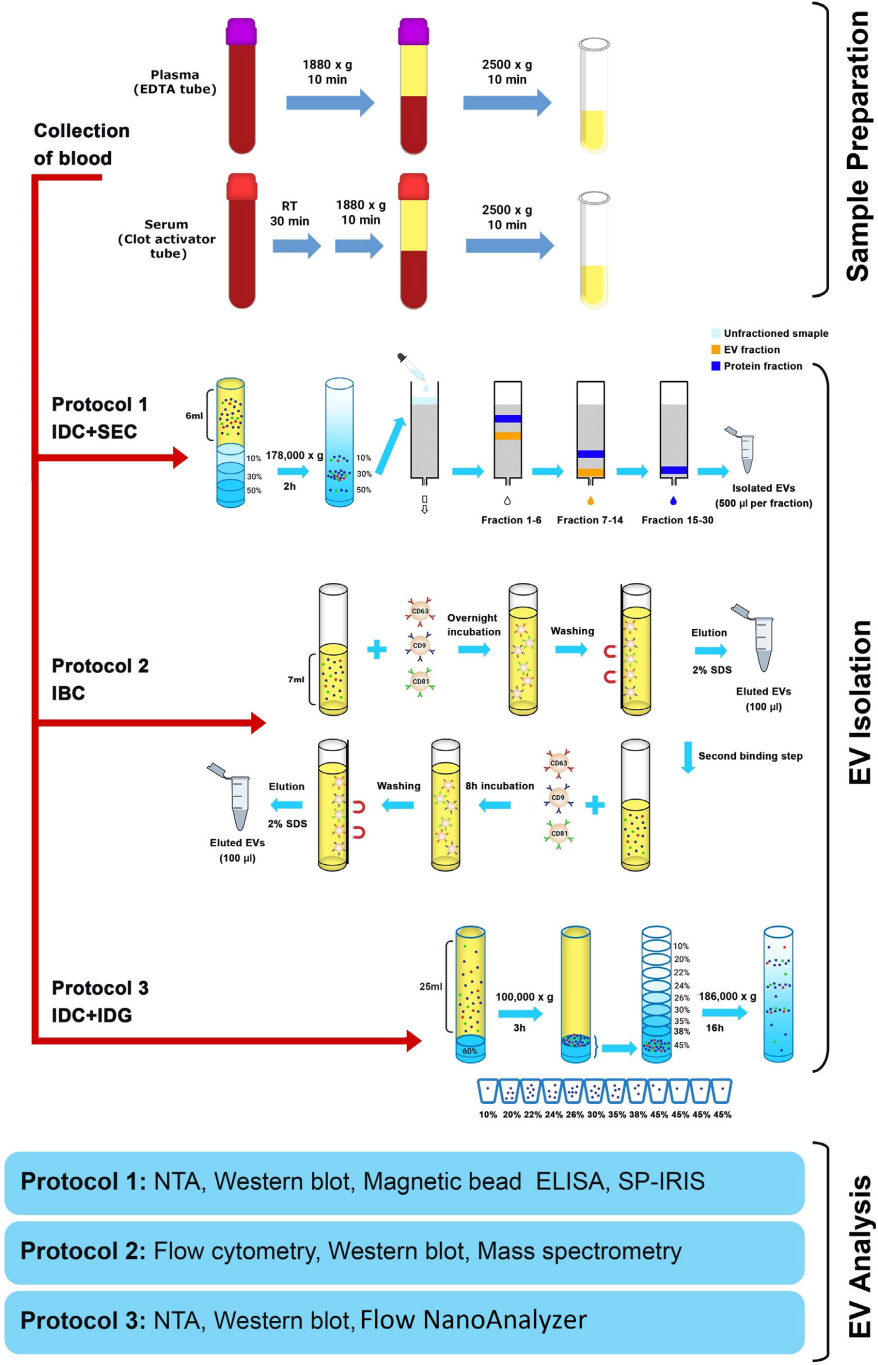
Schematic overview of the experimental workflow. Plasma and serum were collected, and three different protocols were used to isolate EVs. EDTA, ethylenediaminetetraacetic acid; ELISA, enzyme‐linked immunosorbent assay; EV, extracellular vesicle; IBC, immuno‐bead capturing; IDC, iodixanol density cushion; IDG, iodixanol density gradient; NTA, nanoparticle tracking analysis; SDS, sodium dodecyl sulfate; SEC, size exclusion chromatography; SP‐IRIS, single‐particle interferometric reflectance imaging sensing

Peripheral blood was collected from healthy donors in the morning after overnight fasting. A total of 40 ml of peripheral blood was collected from each healthy donor into K2E EDTA (VACUETTE^®^, no. 455036), ACD‐A (VACUETTE^®^, no. 455055) and CAT serum clot activator tubes (VACUETTE^®^, no. 455009). The first few millilitres of blood were drawn into a tube that was discarded afterward. The blood collection tubes were gently inverted 10 times to ensure proper mixing of the tube's additive with the blood. The blood collection tubes were handled directly upon collection and were not transported because blood collection, plasma/serum preparation and EV isolation were carried out in the same laboratory. To prepare plasma for the majority of the study, the whole blood samples in EDTA and ACD‐A tubes were centrifuged at 1880 × *g* at room temperature (RT) for 10 min. The plasma was then transferred to a new tube and centrifuged at 2500 × *g* for 10 min at RT. For one experiment in the end of the study, this plasma isolation protocol was compared to the plasma isolation protocol recommended by the International Society on Thrombosis and Haemostasis (ISTH) (Coumans et al., 2017). Following the ISTH‐recommended protocol, the whole blood samples in EDTA and ACD‐A tubes were centrifuged at 2500 × *g* at RT for 15 min. The plasma was then transferred to a new tube and centrifuged at 2500 × *g* for 15 min at RT again. For serum, the blood was collected into clot activator tubes. The serum was then prepared by incubating whole blood at RT for 30 min to allow clotting before being centrifuged at 1880 × *g* for 10 min at RT. The serum was finally transferred to a new tube and centrifuged at 2500 × *g* for 10 min at RT. The remaining platelets were counted with a Sysmex automated counter after both the 1880 × *g* and 2500 × *g* centrifugations in EDTA‐, ACD‐plasma and serum (Noderstedt, Germany). Plasma/serum‐derived EVs were isolated directly by several different protocols (Figure 1) after the preparation of plasma and serum was complete, and the plasma and serum samples were not frozen prior to EV isolation. The isolated EVs were then stored at −80°C until further analyses. Samples were collected with the approval of the Regional Ethical Approval Committee in Gothenburg, Sweden (no. 593‐08).

### 2.2 Cell culture and isolation of cell culture‐derived EVs

EVs from the human mast cell line HMC‐1 (a gift from Dr. Joseph Butterfield, Mayo Clinic, Rochester, MN, USA) were used to optimise Protocols 2 and 3. The cells were cultured in Iscove's Modified Dulbecco's Medium (IMDM, HyClone Laboratories, Logan, UT, USA) supplemented with 10% EV‐depleted foetal bovine serum (FBS; Sigma‐Aldrich, St Louis, MO, USA), 2‐mM L‐glutamine (Sigma‐Aldrich), 100 units/ml penicillin (HyClone), 100 μg/ml streptomycin (HyClone) and 1.2 U/ml alpha‐thioglycerol (Sigma‐Aldrich). The cells were grown at 37°C in a 5% CO_2_ humidified incubator. The supplemental FBS was depleted of EVs by ultracentrifugation for 18 h at 118,500 × *g*_avg_ (Type 45 Ti rotor, k‐factor 177, Beckman Coulter, Brea, CA, USA) at 4°C and passed through a 0.22‐μm filter before being added to the media. Cells were passaged every 2–3 days, and cell viability was assessed using trypan blue exclusion methods.

Cell‐conditioned media (600–1200 ml) from 80% confluent HMC‐1 cells grown in T175 cell culture flasks (2 × 10^6^ HMC‐1 cells/ml) was used for EV isolation. To isolate EVs from HMC‐1 cells, the conditioned medium was centrifuged at 300 × *g* for 10 min and at 2000 × *g* for 20 min to eliminate cells and cell debris/larger EVs, respectively. Supernatants were then centrifuged at 16,500 × *g*_avg_ (Type 45 Ti rotor, k‐factor 1274, Beckman Coulter) for 20 min at 4°C and then at 118,500 × *g*_avg_ (Type 45 Ti rotor, k‐factor 177, Beckman Coulter) for 2.5 h at 4°C (100 ml, Quick‐seal Round Top Polypropylene Tube, product no: 345776, Beckman Coulter). The pellets from the two final ultracentrifugation steps were dissolved in PBS and mixed and then stored at −80°C. The isolated EVs from HMC‐1 cells were used for the optimisation step in Protocol 2, while the conditioned media after centrifugation at 300 × *g* was used for optimisation steps in Protocol 3.

### 2.3 Protocol 1 – isolation of EVs by an iodixanol density cushion and SEC

We previously developed a method for isolating EVs from plasma samples (Karimi et al., 2018), and this was applied in the current study for comparing the composition of EVs in serum and plasma. Briefly, a 50% iodixanol (OptiPrep™, Sigma Aldrich) working solution was prepared and used to further prepare 30% and 10% iodixanol solutions. Next, 6‐ml plasma or serum was layered on top of 2‐ml 50%, 2‐ml 30% and 2‐ml 10% iodixanol solutions (13.2 mL, Open‐Top Thinwall Ultra‐Clear Tube, product no: 344059, Beckman Coulter) before being ultracentrifuged at 178,000 × *g*_avg_ for 2 h at 4°C (SW 41 Ti rotor, k‐factor 143.9, Beckman Coulter). A visible EV‐enriched band with a volume of 1 ml was collected from the 30% and 10% interface and then loaded onto a home‐made SEC column packed with Sepharose CL‐2B (GE Healthcare, Uppsala, Sweden) in a Telos SPE column (Kinesis, Cambridgeshire, UK) as previously described in Ax et al. (2020) and Karimi et al. (2018). The collection of 0.5‐ml fractions immediately begun when the sample was added. In total, 30 fractions of 0.5 ml were eluted and collected using 0.2‐μm‐filtered PBS as the elution buffer.

### 2.4 Protocol 2 – isolation of EVs by immune bead capturing

In the current study, we developed a magnetic bead‐based method for isolating subpopulations of EVs based on three different types of tetraspanins, including CD63, CD9 and CD81. Isolation was carried out using magnetic beads coupled with antibodies against CD63 (Thermo Fisher Scientific, catalogue no. 10606D), CD81 (Thermo Fisher Scientific, catalogue no. 10622D) or CD9 (Thermo Fisher Scientific, catalogue no. 10620D). This protocol was used to analyse the captured EVs by flow cytometry (EVs still bound to the beads) and by mass spectrometry and Western blot. For the latter two assays, EVs were eluted off the beads prior to analysis. The ratio of starting material to bead concentration, as well as some other steps, were different between isolating the EVs for flow cytometry and isolating EVs for mass spectrometry and Western blot, and these protocols are therefore reported separately here.

For isolating EVs to be analysed by mass spectrometry and Western blot, anti‐CD63, anti‐CD81 and anti‐CD9‐coupled magnetic beads were incubated overnight at 4°C on a rotating shaker in individual tubes with fresh plasma/serum samples at a ratio of 7‐ml plasma or serum per 3.7 × 10^6^ anti‐CD63 beads and per 9.9 × 10^6^ anti‐CD9 and anti‐CD81 beads. Because the anti‐CD63 beads were larger in size than the anti‐CD9 and anti‐CD81 beads, this ratio was calculated so that the total surface areas of the beads were the same. The bead‐EV complexes were collected using a magnetic separator, and the non‐bead‐bound plasma/serum was subjected to a second binding process by adding the same amount of new anti‐CD63, anti‐CD81 and anti‐CD9‐coupled magnetic beads and incubating for another 8 h at 4°C with rotation. This second round of binding was performed to ensure that all EVs present in plasma and serum were captured and that the number of beads was not a limiting factor. The bead‐EV complexes from the first and second binding processes were then washed three times with 700 μl of 0.001% Tween in PBS prior to the elution step. For the mass spectrometry and Western blot analysis, EVs were eluted off the magnetic beads by adding 100‐μl 2% SDS. Lastly, the eluted EVs from the first and second binding step were mixed (a final volume of 200 μl) and used for Western blot and mass spectrometry analysis.

To optimise the beads for flow cytometry analysis, the protocol was slightly altered. Importantly, to ensure that a signal could be detected, the ratio of starting material to beads was altered. Briefly, 1200 ‐μl plasma and serum were incubated with 1.3 × 10^5^ anti‐CD9 and anti‐CD81 beads or 5 × 10^4^ anti‐CD63 magnetic beads for 40 min at RT on a rotating shaker. The bead‐EV complexes were collected using a magnetic separator and were further washed with 1% EV‐depleted FBS in PBS and incubated with human IgG (Sigma‐Aldrich) for 15 min at 4°C. The EV‐bead complexes were washed twice and then incubated with anti‐CD9‐PE (clone M‐L13), anti‐CD63‐PE (clone H5C6), anti‐CD81‐PE (clone JS‐81) or the corresponding isotype control (all antibodies were from BD Biosciences, San Jose, CA, USA) for 40 min at RT with gentle agitation. Lastly, the samples were washed three times with 1% EV‐depleted FBS in PBS, and 10,000 events were acquired on a BD FACSVerse™ flow cytometer running BD FACSSuite™ software (BD Bioscience) and analysed with FlowJo Software (Tree Star, Inc.) The data are presented both as histograms in the figures and as mean fluorescence intensity (MFI; median) in a table.

### 2.5 Evaluation of the capacity to detect CD63 and CD81 on HMC‐1 EVs captured on anti‐CD81 and anti‐CD63 beads

To rule out that the lack of a signal for some markers in plasma and serum was not due to steric hindrance or competition for the same epitope by the capturing and detecting antibody, we tested the beads and antibodies on HMC‐1 EVs. EVs isolated from HMC‐1 cells (a mixture of EVs from the 16,500 × *g* and 118,000 × *g* centrifugations) were incubated with anti‐CD63, anti‐CD9 and anti‐CD81‐coated beads (all from Thermo Fisher Scientific) under gentle rotation at RT for 15 min, followed by overnight incubation at 4°C under gentle rotation. For the anti‐CD63 beads, 30‐μg EV protein/5 × 10^4^ beads/antibody was used, and for anti‐CD81 and anti‐CD9 beads, 30‐μg EV protein/1.3 × 10^5^ beads/antibody was used. The bead‐EV complexes were collected using a magnetic separator and were further washed with 1% EV‐depleted FBS in PBS and incubated with human IgG (Sigma‐Aldrich) for 15 min at 4°C. The EV‐bead complexes were washed twice and then incubated with anti‐CD9‐PE (clone M‐L13), anti‐CD63‐PE (clone H5C6), anti‐CD81‐PE (clone JS‐81) or the corresponding isotype control (all antibodies were from BD Biosciences, San Jose, CA, USA) for 40 min at RT with gentle agitation. Lastly, the samples were washed three times with 1% EV‐depleted FBS in PBS, transferred to FACS tubes (5‐ml Round Bottom Polystyrene Test Tube, product no: 352052, Falcon) and 10,000 events were acquired on a BD FACSVerse™ flow cytometer running BD FACSSuite™ software (BD Bioscience) and analysed with FlowJo Software (Tree Star, Inc.).

### 2.6 Elution of HMC‐1‐derived EVs from beads

To develop the protocol for eluting EVs off of the beads, four different elution buffers were first tested on HMC‐1‐derived EVs. A glycine buffer (Glycin‐HCl, 0.5 M, pH 2.5), a commercial buffer (Pierce™ Gentle Ag/Ab Elution Buffer, pH 6.6, Thermo Fisher Scientific), a high salt/low pH buffer (acid washing buffer, 10‐mM HEPES, 10‐mM MES,120‐Mm NaCl, 0.5‐mM MgCL2, 0.9‐mM CaCl2) or 2% SDS was used for eluting the HMC‐1‐derived EVs off of the magnetic beads. PBS was used as a negative control. For a direct comparison of different elution buffers, EVs isolated from HMC‐1 cells (a mixture of EVs from the 16,500 × *g* and 118,000 × *g* centrifugations) were incubated with anti‐CD63, anti‐CD9 and anti‐CD81‐coated beads (all from Thermo Fisher Scientific) under gentle rotation at RT for 15 min, followed by overnight incubation at 4°C under gentle rotation. For the anti‐CD63 beads, 30‐μg EV protein/5 × 10^4^ beads/antibody was used, and for anti‐CD81 and anti‐CD9 beads 30‐μg EV protein/1.3 × 10^5^ beads/antibody was used. The bead‐EV complexes were then washed three times with 0.001% Tween in PBS and processed using the different elution buffers. Elution was performed by adding 100 μl of the different buffers to the beads, vortexing briefly, and incubating with gentle agitation for one hour at RT. The magnetic beads were collected using the magnetic separator, and the eluted EVs were collected by collecting the supernatant in which the EVs were suspended. The EVs were subjected to Western blot and the beads were subjected to flow cytometry to monitor the amount of EVs remaining bound to the beads after the elution.

### 2.7 Flow cytometry analysis of the beads to evaluate the effect of the elution buffers

The collected anti‐CD63 beads from the buffer experiment were washed twice with a buffer containing 1% EV‐depleted FBS in PBS, incubated with human IgG (Sigma‐Aldrich) for 15 min at 4°C and then washed again twice. The bead‐EV complexes were then incubated with anti‐CD63‐PE (clone H5C6; BD Biosciences) for 40 min at RT under agitation in the dark. Next, the beads were washed twice before analysis on a BD FACSVerse™ flow cytometer running BD FACSSuite™ software (BD Biosciences) with 10,000 events being acquired. Data were analysed with the FlowJo Software (TreeStar, Inc., Ashland, OR, USA).

### 2.8 Protocol 3 – isolation of EVs by an iodixanol density cushion and iodixanol density gradient (IDC+IDG)

A total of 36‐ml of HMC‐1 media (used in the optimisation step) or 25‐ml plasma or serum was added to an ultracentrifuge tube and then 2‐ml of 60% iodixanol was carefully laid at the bottom of the tube (39‐ml, Quick‐seal^®^ Round Top Polypropylene Tube, product no: 340414, Beckman Coulter) using a Hamilton blunt point 4‐inch needle (Hamilton Company, Reno, NV, USA). The sample was then centrifuged at 100,000 × *g*_avg_ (Type 70 Ti rotor, k‐factor 157, Beckman Coulter, Brea, CA, USA) for 3 h at 4°C. A blunt point needle was then used to aspirate 3‐ml from the bottom of the tube, which included the 2‐ml of iodixanol and the 1‐ml of the solution on top of it, which produced a mixture of the sample in 40% iodixanol. This 3‐ml solution was further fractioned on an iodixanol density gradient (IDG). Briefly, a total of 3‐ml concentrate was mixed with 1‐ml of 60% iodixanol and laid at the bottom of the tube, and 1‐ml layers of 38%, 35%, 30%, 26%, 24%, 22%, 20% and 10% iodixanol were carefully overlaid forming a discontinuous gradient. The gradient was then centrifuged at 186,000 × *g*_avg_ (SW 41 Ti, k‐factor 138.0, Beckman Coulter, Brea, CA, USA) for 16 h at 4°C. After centrifugation, 1‐ml fractions were collected from the top to the bottom of the tube.

### 2.9 Nanoparticle tracking analysis (NTA)

The particle concentration of the EVs isolated by Protocols 1 and 3 was measured by NTA using a ZetaView^®^ PMX 120 device and ZetaView^®^ PMX 110 device, respectively (Particle Metrix, Meerbusch, Germany). Briefly, EV‐enriched preparations from Protocols 1 and 3 were thawed immediately before measurements and were re‐suspended in 1 ml of 0.2‐μm‐filtered PBS (50–1000‐fold dilution) before the measurement. Samples were manually injected into the instrument using a 1‐ml syringe. The measurement was carried out at all 11 different positions, and the video quality was set to medium and the camera sensitivity was set to 80. Data were analysed using the ZetaView^®^ analysis software (versions 8.05.11, 8.05.12 and 8.2.30‐1) with a minimum size of 10, a maximum size of 1000 and a minimum brightness of 30.

### 2.10 Protein measurement of EV preparations

The protein concentration of plasma and serum‐derived EVs isolated by Protocol 2 was measured using a Micro Pierce BCA™ Protein Assay Kit (Thermo Fisher Scientific, Waltham, MA) according to the manufacturer's instructions. The protein concentration was measured in the supernatant after the EVs had been eluted off the beads. The protein concentration in plasma and serum‐derived EVs isolated by Protocols 1 and 3 was determined using Qubit (Thermo Fisher Scientific) according to the manufacturer's protocol.

### 2.11 Western blotting

Equivalent micrograms of protein (3 μg) or volumes (36 μl) of samples from the different protocols lysed in 20‐mM Tris‐HCl 1% SDS were loaded and separated on precast 4–20% polyacrylamide Mini‐PROTEAN TGX gels (Bio‐Rad Laboratories, Hercules, CA). In Figure 8d, platelets were used as a positive control. The platelets were isolated by collecting blood in EDTA tubes as described above. The blood was centrifuged at 200 × *g* for 20 min, the plasma was moved to a new tube and centrifuged at 2500 × *g* for 15 min. The pelleted cells were re‐suspended in 500‐μl PBS, and 20 μl was loaded on the gel. The separation was carried out under the recommendations for each individual antibody, including reducing conditions for anti‐calnexin, anti‐flotillin‐1, anti‐mitofillin, anti‐apolipoprotein A1, anti‐CD235a and anti‐p‐selectin and non‐reducing conditions for anti‐CD63, anti‐CD41a, anti‐ADAM10, anti‐CD9 and anti‐CD81 antibodies. After transferring to PVDF membranes (Bio‐Rad Laboratories, Hercules, CA, USA), the membranes were blocked with 5% Blotting Grade Blocker Non‐Fat Dry Milk in TBS containing 0.1% Tween‐20 (TBST) for 1 h at RT and then incubated with the following primary antibodies diluted in 2.5% Blotting Grade Blocker Non‐Fat Dry Milk in TBST at 4°C overnight: anti‐calnexin (1:1,000 dilution, clone C5C9, Cell Signalling Technology, Leiden, the Netherlands), anti‐flotillin‐1 (1:1,000 dilution, clone EPR6041, Abcam, Cambridge, UK), anti‐CD63 (1:1,000 dilution, clone H5C6, BD Biosciences), anti‐CD9 (1:1,000 dilution, clone MM2/57, Millipore, Darmstadt, Germany), anti‐CD81 (1:1,000 dilution, clone M38, Abcam), anti‐mitofilin (1:500 dilution, polyclonal, Invitrogen, Carlsbad, CA, USA), anti‐ADAM10 (1:500 dilution, clone 163003, R&D System, Minneapolis, MN, USA), anti‐apolipoprotein A1 (1:1,000, GTX112692, Genetex, San‐Antonio, TX, USA), anti‐CD41a (1:1,000, clone D8V7H, Cell Signalling Technology), anti‐235a (1:1,000, clone JC159, Thermo Fisher Scientific) and anti‐p‐selectin (1:1,000 dilution, polyclonal, Abcam). The membranes were washed three times in TBST and were then incubated with the appropriate HRP‐conjugated secondary antibodies diluted 1:5,000 in 2.5% Blotting Grade Blocker Non‐Fat Dry Milk in TBST. The secondary antibodies were donkey anti‐rabbit IgG HRP‐linked F(ab)2 fragment (1:5,000 dilution; catalogue no. NA9340V), and sheep anti‐mouse IgG HRP‐linked F(ab)2 fragment (1: 5,000 dilution; catalogue no. NA9310V) (both from GE Healthcare, Buckinghamshire, UK) and were added for 1 h at RT. The membranes were then washed four times for 5 min each in TBST and analysed with the SuperSignal West Femto maximum sensitivity substrate (Thermo Fisher Scientific) on a ChemiDoc Imaging System (Bio‐Rad Laboratories, Hercules, CA, USA).

### 2.12 Magnetic enzyme‐linked immunosorbent assay (ELISA)

The ExoPheno™ platform was used to measure the co‐localisation of EV markers on isolated plasma and serum EVs. The technique captures EVs based on a selection of EV markers and measures the relative abundance of the markers on the captured EVs in an ELISA (Dias et al.). First, fractions 9 and 10 from Protocol 1 were mixed for each serum and plasma sample (*n* = 3), and 11–62 μl of the sample mix was diluted in assay reaction buffer (filtered 1× PBS, 0.02% Tween, 0.1% BSA, pH 7.4). Next, 100 ng of each detection antibody was added to each reaction mixture, that is, one detection antibody per reaction, and incubated for 45 min. The detection antibodies used were anti‐CD9 biotin (MEM‐61, Abcam), anti‐CD41 biotin (MEM‐06 Antibodies‐online GmbH, Aachen, Germany) or isotype IgG1 biotin (MOPC‐21 Abcam). In parallel, the capturing antibodies were coupled to the beads. Briefly, 0.2 mg of the anti‐CD63 (TS63 Abcam), anti‐CD9 (P1/33/2 Abcam) and anti‐CD81 (TS81 Abcam) antibodies were coupled to Dynabeads^®^ M‐270 Epoxy according to the manufacturer's guidelines and stored at 4°C until use. Next, 7.5 μl (75 μg) of either the anti‐CD9, anti‐CD81 or anti‐CD63 magnetic beads were added to the serum and plasma samples. The reaction with the magnetic beads was performed on a 96‐well plate, and mixing was performed every 30 min with an Opentrons OT‐2 automatic liquid handler. After 3‐h incubation, the beads were collected on a Magnetic Stand‐96 and washed four times in washing buffer (filtered 1× PPS, 0.02% Tween, 0.1% BSA, pH 7.4). On the magnetic stand, the washing buffer was removed and 200‐μl streptavidin‐HRP (Thermo Fisher) was added and incubated with agitation for 30 min. Unattached Streptavidin‐HRP was washed away, and 3,3′,5,5′‐tetramethylbenzidine (TMB) was added and incubated for 30 min. Finally, stop solution was added at a proportion of 1:1 to TMB and mixed. The colour change from the developed product was measured on a microplate reader (BMG SPECTROstar Nano Plate Reader) and correlated with the analyte level. A calibration curve with known concentrations of streptavidin poly‐HRP was made to derive the relative EV biomarker expression (data not shown).

### 2.13 Single‐particle interferometric reflectance imaging sensing (SP‐IRIS)

Fraction 9 from Protocol 1 was analysed with the ExoView™ Plasma Tetraspanin kit and an ExoView™ R100 (NanoView Biosciences, Boston, MA) according to the manufacturer's instructions. The ExoView™ Plasma tetraspanin kit captured the EVs with anti‐CD63 (clone H5C6), anti‐CD81 (clone JS‐81), anti‐CD9 (HI9a) and anti‐CD41a (clone HIP8), with mouse IgG as the negative control. Briefly, 50 μl of the sample was mixed with 50 μl of incubation solution (1:1 dilution), and 35 μl of the diluted samples were added directly to the chip and incubated at RT for 16 h. The samples were then subjected to immuno‐fluorescence staining using fluorescent antibodies (CD9‐CF488 (clone HI9a), CD63‐CF647 (clone H5C6) and CD81‐CF555 (clone JS‐81), provided in the ExoView™ Plasma Tetraspanin kit) before they were washed and scanned using an ExoView™ R100 imaging system. The data were analysed using the Nanoviewer analysis software version 2.8.10.

### 2.14 Flow NanoAnalyzer

A pool of the 20% and 22% fractions from Protocol 3 was analysed using a Flow NanoAnalyzer (NanoFCM Inc., Xiamen, China) according to the manufacturer's instruction. Briefly, the Flow NanoAnalyzer was aligned using polystyrene quality control reference beads (NanoFCM Inc.). Furthermore, two types of beads were used to be able to determine the size of the EVs (Silica nanospheres; 68–155 and 155–850 nm; NanoFCM Inc.). First, unstained samples were analysed to determine the concentration and the size of the EVs. As the particle number was in the 2000–12,000 events/min range recommended by the company, no dilution was needed for the samples. For the immunofluorescent staining, a volume of 2 μl of FITC‐labelled anti‐CD9 (clone M‐L13) and 2‐μl PerCPCy5.5‐labeled anti‐CD41a (Clone HIP8) was mixed and then 1 μl of this antibody mix was added to 4 μl of EV sample (final dilution of antibodies was 10× (0.025 μg), and both antibodies were from BD Biosciences) (0.6‐ml Maxyclear SnapLock Microcentrifuge Tubes, product no: MCT‐060‐C, Axygen). Additionally, 1 μl of the antibody mix was also added to 4‐μl PBS as a control. The mixture was incubated in the dark for 40 min at RT. First 45‐μl PBS was added to the 5‐μl EV‐antibody mix and the 5‐μl PBS‐antibody mix (10× dilution), then 10 μl was taken from each of the samples and the control and 40‐μl PBS was added (final dilation: 50×). All samples (plasma and serum EVs) and the control (PBS+antibodies) were acquired for 1 min on the instrument. The instrument parameters of the Flow NanoAnalyzer analysis were set as follows: laser, 10 mW, 488 nm; SS decay, 10%; sampling pressure, 1.5 kPa; time to record, 1 min, a 525/40 filter for FITC and a 670/30 filter for PerCPCy5.5. Particle concentration and size distribution were calculated using the NanoFCM software (NF profession V1.0)

### 2.15 Sample preparation and digestion for mass spectrometry analysis

The proteomic analysis was performed at The Proteomics Core Facility at Sahlgrenska Academy, Gothenburg University. Samples were isolated with Protocol 2, and in total 40 μg per sample was used, with three biological replicates for each EV subpopulation. A reference pool was constructed consisting of 4.5 μg from each of the nine samples. The samples and reference pool were digested with trypsin using the suspension trapping (S‐Trap™, Protifi) spin column digestion method according to the manufacturer´s instructions. Samples in 2% SDS were reduced with 5‐mM dithiothreitol (56°C, 30 min) and alkylated using 10‐mM methyl methanethiosulfonate (RT, 20 min). Samples were acidified with phosphoric acid, mixed with S‐Trap binding buffer (90% MeOH in 100‐mM triethylammonium bicarbonate (TEAB)), transferred to S‐Trap™ micro spin columns and washed several times with binding buffer. Digestion was performed in 50‐mM TEAB at 37°C by addition of 1‐μg Pierce MS‐grade trypsin (Thermo Fisher Scientific) and incubated overnight in a humidified chamber. Peptides were eluted by centrifugation in three steps – (1) 50‐mM TEAB, (2) 0.2% formic acid and (3) 50% acetonitrile in 0.2% formic acid – and the eluates were pooled. The peptides were dried in a vacuum concentrator, resolved in 50‐mM TEAB, and labelled using TMT 10‐plex isobaric mass tagging reagents (Thermo Scientific) according to the manufacturer's instructions. The combined purified samples were pre‐fractionated into 40 fractions with basic reversed‐phase chromatography using a Dionex Ultimate 3000 UPLC system (Thermo Fischer Scientific). Peptide separations were performed using a reversed‐phase XBridge BEH C18 column (3.5 μm, 3.0 × 150 mm, Waters Corporation) and a linear gradient from 3% to 40% solvent B over 18 min followed by an increase to 100% B over 5 min and 100% B for 5 min at a flow of 400 μl/min. Solvent A was 10‐mM ammonium formate buffer at pH 10.00 and solvent B was 90% acetonitrile and 10% 10‐mM ammonium formate at pH 10.00. The fractions were concatenated into 20 fractions, dried and reconstituted in 3% acetonitrile in 0.2% formic acid.

### 2.16 nanoLC‐MS/MS analysis and database search

Each fraction was analysed on an Orbitrap Fusion™ Lumos™ Tribrid™ mass spectrometer (Thermo Fisher Scientific) interfaced with an nLC 1200 liquid chromatography system. Peptides were trapped on an Acclaim Pepmap 100 C18 trap column (100 μm × 2 cm, particle size 5 μm, Thermo Fischer Scientific) and separated on an in‐house constructed analytical column (350 mm × 0.075 mm I.D.) packed with 3‐μm Reprosil‐Pur C18‐AQ particles (Dr. Maisch, Germany) using a linear gradient from 5% to 33% B over 77 min followed by an increase to 100% B for 3 min and 100% B for 10 min at a flow of 300 nl/min. Solvent A was 0.2% formic acid in water and solvent B was 80% acetonitrile in 0.2% formic acid. Precursor ion mass spectra were acquired at 120,000 resolution, and MS/MS analysis was performed in a data‐dependent multinotch mode where CID spectra of the most intense precursor ions were recorded in the ion trap at a collision energy setting of 35 for 3 s (‘top speed’ setting). Precursors were isolated in the quadrupole with a 0.7 *m*/*z* isolation window, charge states 2–7 were selected for fragmentation, and dynamic exclusion was set to 45 s and 10 ppm. MS3 spectra for reporter ion quantitation were recorded at 50,000 resolution with HCD fragmentation at a collision energy of 65 using the synchronous precursor selection.

The data files for each set were merged for identification and relative quantification using Proteome Discoverer version 2.4 (Thermo Fisher Scientific). The search was against human Swissprot Database version March 2019 (Swiss Institute of Bioinformatics, Switzerland) using Mascot 2.5 (Matrix Science) as the search engine with a precursor mass tolerance of 5 ppm and a fragment mass tolerance of 0.6 Da. Tryptic peptides were accepted with zero missed cleavages, variable modifications of methionine oxidation and fixed cysteine alkylation, and TMT‐labelled modifications of N‐terminal and lysine were selected. Percolator was used for PSM validation with a strict FDR threshold of 1%, and the quantified proteins were filtered at 5% FDR and grouped by sharing the same sequences in order to minimise redundancy. TMT reporter ions were identified in the MS3 HCD spectra with a 3‐mmu mass tolerance, and the TMT reporter intensity values for each sample were normalised within Proteome Discoverer 2.4 based on the total peptide amount. Only peptides unique for a given protein were considered for the relative quantification. A reference sample made from a mix of all the samples was used as the denominator for calculating the ratios.

### 2.17 Spike‐in experiment with mCherry‐engineered EVs

The mCherry‐engineered EVs were a kind gift from Elisa Lázaro‐Ibáñez, Pharmaceutical Science, AstraZeneca, Gothenburg, Sweden, and they have previously been described in detail in Lázaro‐Ibáñez et al. (Lázaro‐Ibáñez et al., 2021). The mCherry‐engineered EVs were shown to be 126–154 nm as measured by NTA and <100 nm in size as determined by negative stain and electron microscopy (Lázaro‐Ibáñez et al., 2021). Briefly, Expi293F cells were transiently transfected with a pEBNAZ DNA plasmid coding for mCherry fused to the C‐terminus of human CD63. The sequence was codon‐optimised for human expression by GeneScript (CD63 protein sequence P08962 and mCherry protein sequence X5DSL3). Large EVs were removed by a 20,000 × *g* centrifugation before small EVs were isolated by centrifugation at 100,000 × *g* for 120 min. The small EVs were further purified on a high‐resolution IDG (Lázaro‐Ibáñez et al., 2021). The concentration of the mCherry‐engineered EVs was determined to be 10^9^/μl with NTA. Fifteen microliters of mCherry‐engineered EVs were added to the EDTA (plasma tube) and clot activator (serum tube) tubes immediately after blood was collected and gently inverted to mix. Blood was incubated for 30 min for both the EDTA and clot activator tubes before being centrifuged at RT for 10 min at 1880 × *g*. Plasma and serum were then transferred to new tubes and centrifuged again at 2500 × *g* for 10 min at RT. The plasma and serum were used immediately for EV isolation by Protocol 1. An aliquot of 90 μl from each step was kept, and the fluorescence signal (Ex 590 nm, Em 645 nm) was measured on a Varioskan™ LUX multimode microplate reader (Thermo Fisher Scientific).

### 2.18 Literature search for methods for blood collection and handling in published EV studies

The EV‐TRACK knowledgebase was searched for all human plasma studies on the 4th of October 2021, and 547 experiments were identified (Consortium et al., 2017). As some experiments belonged to the same studies, we ended up with 244 individual studies in the end. These 244 studies were further identified via PubMed, and their methods sections with a focus on the blood collection and handling were analysed manually. The information identified for all studies was which type of anti‐coagulant was used and how the blood/plasma was centrifuged prior to freezing and EV isolation.

### 2.19 Bioinformatics and statistical analysis

Where appropriate, data are expressed as the mean with either individual values or SEM shown. Statistical analysis was performed with Student's non‐paired *t*‐test. Qlucore Omics Explorer (Qlucore, Lund, Sweden) was used for the principal component analysis. DAVID was used to determine the associated cellular components (https://david.ncifcrf.gov/) (Huang da et al., 2009a,b).

### 2.20 Data availability

The MS proteomics data have been deposited to the ProteomeXchange Consortium via the PRIDE partner repository with the dataset identifier PXD026863 (Deutsch et al., 2020; Perez‐Riverol et al., 2019). We have submitted all relevant data from our Protocol 1 to the EV‐TRACK knowledgebase (EV‐TRACK ID: EV220003) (Consortium et al., 2017) as this was the protocol that was used for the majority of the experiments.

## 3 RESULTS

### 3.1 CD9 and CD41a‐containing EVs are more prominent in EDTA‐plasma compared to serum

**FIGURE 2:**
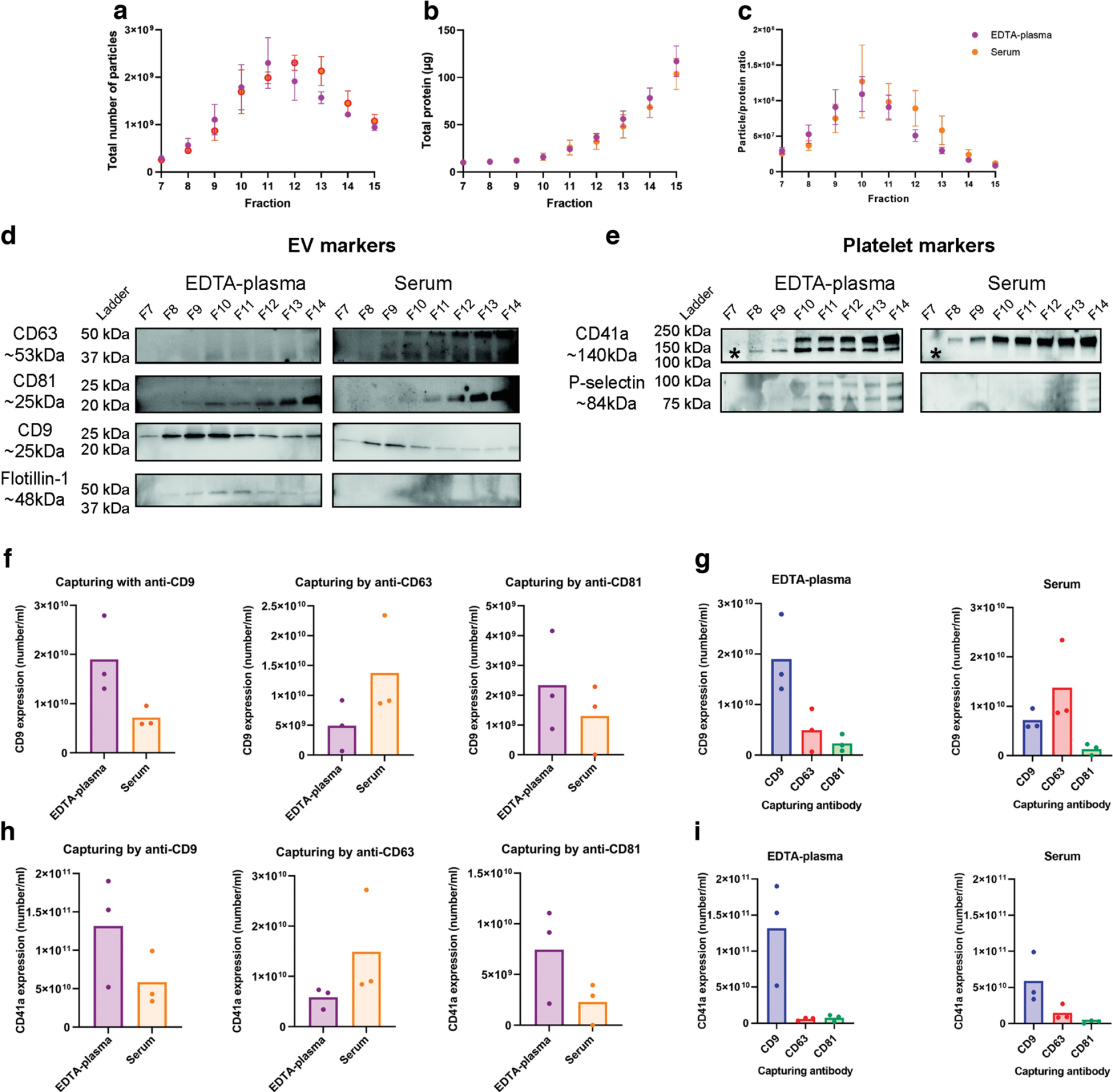
CD9^+^ and CD41a^+^ extracellular vesicles (EVs) are more prominent in EDTA‐plasma compared to serum. EVs were isolated with Protocol 1 starting with 6‐ml plasma or serum. a,b. Concentrations of particles (a) and proteins (b) in the SEC fractions were determined with NTA and Qubit, respectively. Data are presented as the total amount of particles or proteins in fractions 7–15. *N* = 4, and the results are presented as the average ± SEM. c. The particle to protein ratio based on the values from panels a and b. *N* = 4, and the results are presented as the average ± SEM. d,e. The presence of the EV markers CD63, CD81, CD9 and flotillin‐1 (d) and the platelet markers CD41a and p‐selectin (e) were determined in SEC fractions 7–14 (36 μl/fraction) with Western blotting. CD41a is detected at 140 kDa, which is the lower band only detected in plasma and highlighted with *. F–I. The presence of the EV marker CD9 (f,g) and the platelet‐marker CD41a (h,i) was determined on EVs from pooled fractions 9 and 10 from EDTA‐plasma and serum, respectively, captured by anti‐CD9, anti‐CD63 and anti‐CD81 magnetic bead ELISA. *N* = 3. f and g show the same data but displayed either per protein or per biofluid. EVs were captured with anti‐CD9, anti‐CD63 or anti‐CD81 beads and CD9 was detected. h and i show the same data but displayed either per protein or per biofluid. EVs were captured with anti‐CD9, anti‐CD63 or anti‐CD81 beads, and CD41a was detected. No significance was observed for G and I when paired Student's *t*‐test was used

We previously described increased plasma EV purity when isolated with a combination of an iodixanol density cushion (IDC) and SEC, resulting in reduced quantities of lipoproteins in the EV‐enriched sample (Karimi et al., 2018). Here we applied this protocol (IDC+SEC, hereafter called Protocol 1, Figure 1) to compare EVs isolated from plasma and serum. All plasma and serum samples were paired (collected from the same person at the same time). EVs were first isolated from 6 ml of fresh EDTA‐plasma or serum with our previously published Protocol 1. The EV‐enriched band in the interphase between 10% and 30% iodixanol (1 ml in volume) was collected and loaded onto individual SEC columns, and subsequent NTA confirmed that the majority of the particles were found in fractions 9–14 in both EDTA‐plasma and serum (Figure 2a). Proteins mainly eluted beyond fraction 12 (Figure 2b). No difference in absolute particle number or absolute protein quantity was observed in the SEC fractions (Figure 2a,b). The particle to protein ratio has previously been suggested to be used as a measurement of the purity of isolated EVs (Thery et al., 2018). Our data showed that the highest ratio was detected in fractions 9–11 in both EDTA‐plasma and serum, indicating that these fractions contained the highest relative concentration of EVs (Figure 2c).

Because NTA cannot distinguish between EVs and other particles such as aggregates and lipoprotein particles, we next used more specific measurements that included the detection of several EV markers as well as non‐EV markers. As it was shown that the majority of the EVs were present in fractions 9–11 (Figure 2a,c), further analysis was focused on these and their adjacent fractions. Western blot demonstrated the presence of flotillin‐1 and CD9 in fractions 8–11 in EDTA‐plasma with the strongest signal in fraction 10, while no signal was detected in the serum sample for flotillin‐1 and only weak signals were detected for CD9 (Figure 2d). CD63 and CD81 were present in fractions 9–14, but interestingly they peaked in different fractions (fraction 10 vs. fraction 14, respectively) (Figure 2d). In addition to the traditional EV markers, we also determined the presence of the platelet marker CD41a and the activated platelet marker p‐selectin (Saboor et al., 2013). CD41a and p‐selectin were present in fractions 8–14 and 10–14, respectively, in EDTA‐plasma but were not present in serum, suggesting that platelet‐derived EVs were mainly present in EDTA‐plasma (Figure 2e).

Because Western blot does not reveal whether the analysed markers are present on the same vesicles or on different vesicles, we next utilised magnetic bead ELISAs to further determine the presence of CD41a, CD63, CD9, and CD81 on individual EVs. The magnetic bead ELISA involves capturing EVs on anti‐CD9, anti‐CD63 and anti‐CD81 beads and then further staining for CD9 or CD41a. For this experiment, fractions 9 and 10 were mixed and analysed because they had the highest particle/protein ratio. More CD9/CD9 vesicles were present in the EDTA‐plasma than in serum (Figure 2f), thus validating the Western blot data (Figure 2d). Preferably, we would next have analysed CD63/CD63 and CD81/CD81 EVs to compare the presence of CD63, CD9 and CD81 EVs in plasma and serum, but these combinations are currently not available on this platform. Therefore, we instead determined the presence of CD63/CD9 and CD81/CD9 EVs. Interestingly, CD63/CD9 was more abundant in serum than in EDTA‐plasma, whereas we were unable to detect any difference in CD81/CD9 between the two biofluids (Figure 2f). When the three combinations (CD9/CD9; CD63/CD9; CD81/CD9) were compared within the respective biofluid, it was obvious that CD9^+^/CD9^+^ EVs were the most common EV type in EDTA‐plasma, while CD9^+^/CD63^+^ EVs were the most common EVs in serum (Figure 2g). For both EDTA‐plasma and serum, CD9^+^/CD81^+^ EVs were the least common EV type (Figure 2g). When the platelet‐marker CD41a was used as the detection molecule instead of CD9, more CD9^+^/CD41a^+^ and CD81^+^/CD41a^+^ double‐positive EVs were observed in EDTA‐plasma than in serum (Figure 2h). The opposite was observed for CD63^+^/CD41a^+^ double‐positive EVs, which were more prominent in serum than in EDTA‐plasma (Figure 2h). When these three combinations (CD9/CD41a; CD63/CD41a; CD81/CD41a) were compared within the respective biofluid, it was clear that CD41a was primarily associated with CD9^+^ vesicles and to a much lesser extent with CD63^+^ and CD81^+^ vesicles (Figure 2i).

Together, this argues that more CD9^+^ EVs are present in EDTA‐plasma than in serum, while more CD63^+^ EVs are present in serum than in EDTA‐plasma. Furthermore, CD81^+^ EVs were rarest tetraspanin in both EDTA‐plasma and serum. Importantly, CD41a was mainly associated with CD9^+^ EVs, which suggests that the majority of CD9^+^ vesicles in EDTA‐plasma and serum are derived from platelets.

### 3.2 EVs are not lost during serum collection nor during EV isolation

**FIGURE 3:**
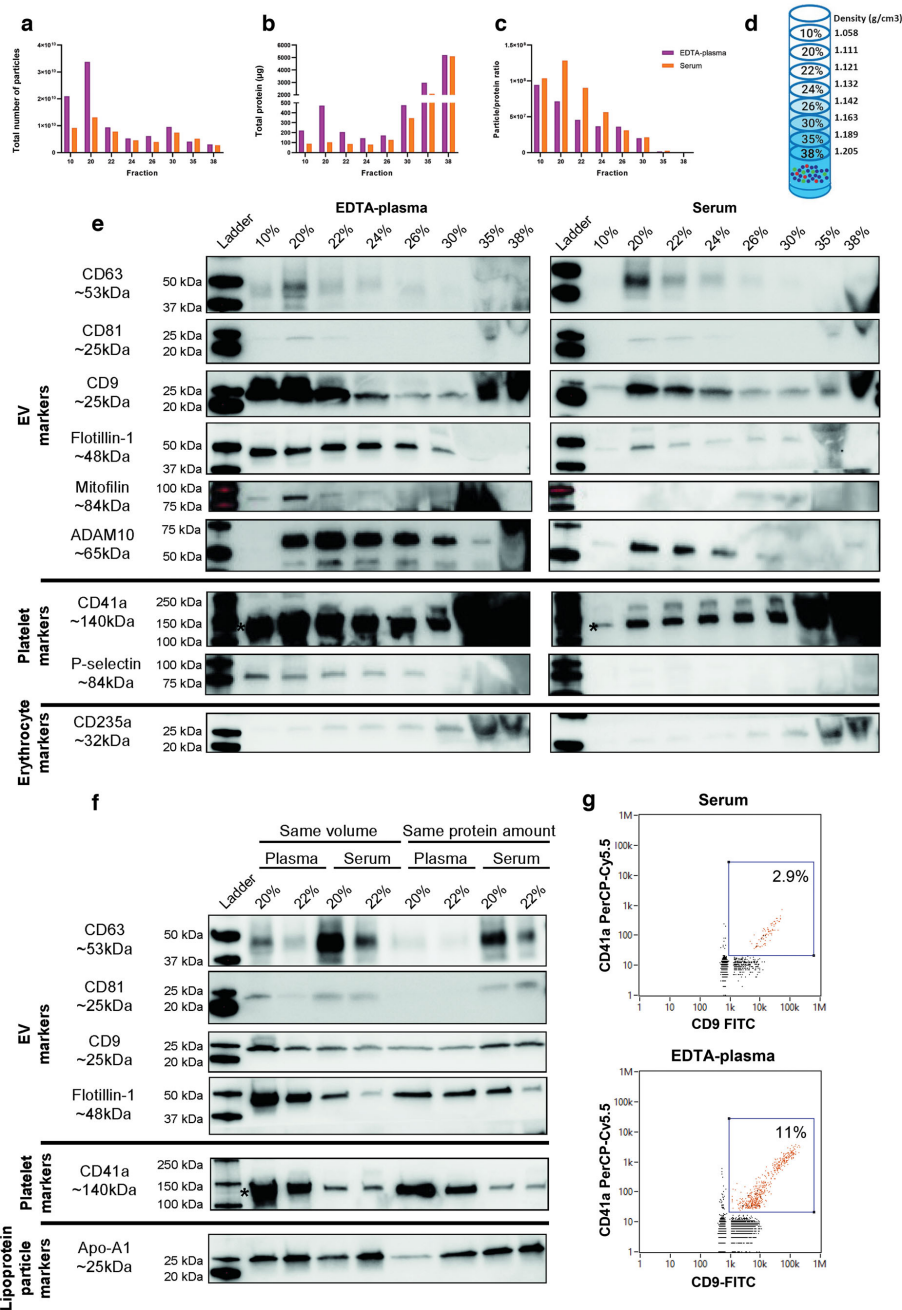
EDTA‐plasma and serum‐derived extracellular vesicles (EVs) have similar densities. EVs were isolated with Protocol 3 (the starting volume of plasma and serum was 25 ml), which is a non‐pelleting protocol consisting of the combination of an iodixanol density cushion and an iodixanol density gradient. a,b. Concentrations of particles (a) and proteins (b) in the density fractions were determined with NTA (ZetaView^®^) and Qubit, respectively. Data are presented as the total amount of particles or proteins in fractions 10%–38%. *N* = 1. c. The particle to protein ratio based on the values from panels a and b. *N* = 1. d. The densities for the different fractions according to the information sheet for iodixanol. e. The presence of the EV markers CD63, CD81, CD9, flotillin‐1, mitofillin and ADAM10; the platelet markers CD41a and p‐selectin; and the erythrocyte marker CD235a were determined with Western blot in the top eight 1‐ml fractions from the iodixanol gradient fractions (36 μl/fraction). CD41a is detected at 140 kDa, which is the lower band highlighted with *. f. The majority of the EVs were present in the 20% and 22% fractions, and we, therefore, loaded these fractions from both serum and plasma onto the same gels, normalised both for volume (36 μl) and protein amount (3 μg). The presence of the EV markers CD63, CD81, CD9 and flotillin‐1; the platelet marker CD41a and the lipoprotein marker Apo‐A1 were determined in the 20% and 22% fractions from the iodixanol gradient. g. The presence of CD41a and CD9 on single EVs was determined with Flow NanoAnalyzer for the pooled 20% and 22% fractions from plasma and serum, respectively. For serum, 2577 events/min were recorded and for plasma 5059 events/min were recorded

To confirm that we did not lose certain EV subpopulations, such as the CD9^+^ population in serum, for example, with our isolation protocol (Protocol 1), we also used an immuno‐bead capturing protocol (IBC, hereafter called Protocol 2, Figure 1) and a non‐pelleting protocol using a combination of IDC and an IDG (IDC+IDG, hereafter called Protocol 3, Figure 1). Hypothetically, the EVs in serum could have a different density compared to the EVs in EDTA‐plasma. To ensure that no EVs were lost from the serum during our isolation using IDC+SEC (Protocol 1), we established a protocol that included vesicles with a broader range of densities (Protocol 3 in Figure 1) compared to the vesicles isolated by the density cushion in Protocol 1. Protocol 3 was modified from Li et al. (Li et al., 2018) and was first established by isolating EVs from conditional cell culture media (Figure S1) because large samples were needed to optimise the protocol and were easier to obtain with cell cultures than EDTA‐plasma and serum. Importantly, this protocol did not contain any pelleting step so as to avoid soluble proteins and other entities being forced together with the EVs or causing EV aggregates. This approach seemingly isolated a majority of cell line‐derived EVs in fractions at densities of 10%–22% iodixanol, with the peak in the 20% fraction (1.11 g/ml, Figure S1A–C). When we implemented Protocol 3 on EDTA‐plasma and serum (starting volume: 25 ml), NTA showed that the majority of the particles were present in the 20% fractions in EDTA‐plasma and serum, and protein measurement showed that a protein peak was visible here as well, although the majority of the proteins were identified at the bottoms of the gradients (Figure 3a,b). The particle to protein ratio was the highest in fractions 10%, 20% and 22% indicating that the purest EVs were present in these fractions (Figure 3c). The densities for the different fractions are shown in Figure 3d according to the information sheet for iodixanol.

When analysed in detail with Western blot, it was evident that CD81 and CD63 had similar trends with both being present in the 20%–24% fractions, with the strongest signal in the 20% fraction and with a tendency to be stronger in serum than in EDTA‐plasma (Figure 3e). CD9 and flotillin‐1 had similar trends with each other with signals in all of the fractions (10%–30%), but the signals were much stronger in EDTA‐plasma than in serum (Figure 3e). No differences were observed for the red blood cell marker CD235a between EDTA‐plasma and serum; however, compared to the other markers, it was present in the denser fractions of 26%–38% (Figure 3e). Interestingly, CD41a was again much stronger in EDTA‐plasma than in serum (Figure 3e), supporting our previous findings with Protocol 1 (Figure 2e,h,i). Because CD41a was present in all densities, its pattern resembled that of flotillin‐1 and CD9 more than the pattern for CD63 and CD81 (Figure 3e). Furthermore, p‐selectin was only detected in fractions 10%–26% in EDTA‐plasma and not at all in serum (Figure 3e), suggesting that EVs in plasma are derived from activated platelets. We and others have recently shown that ADAM10 is enriched in small EVs, while mitofilin is enriched in large EVs (Crescitelli et al., 2020; Kowal et al., 2016). To determine if there was a difference in the presence of small and large EVs in EDTA‐plasma and serum, we also evaluated these markers. ADAM10 was present in both EDTA‐plasma and serum in fractions 20%–30%, with a tendency for stronger bands in EDTA‐plasma than in serum. Mitofilin was only present in EDTA‐plasma and in fraction 20% (Figure 3e). This would suggest that the EVs enriched in EDTA‐plasma compared to serum include both small ADAM10^+^ EVs as well as larger mitofilin^+^ EVs.

To truly be able to compare the EDTA‐plasma and serum samples, we decided to only focus on the 20% and 22% fractions, and we ran these samples on the same gels loaded either based on volume or separately based on protein quantity in order to avoid any gel‐to‐gel differences in the results. This confirmed our previous observations with CD41a and flotillin‐1 being more present in EDTA‐plasma compared to serum, while CD63 and CD81 were more present in serum than in EDTA‐plasma (Figure 3f). No clear differences were observed for CD9 and Apo‐A1 (Figure 3f).

To again evaluate the co‐localisation of CD9 and CD41a, we analysed a mixture of fractions 20% and 22% with a nanoflow cytometer, which allowed us to perform single‐EV analysis. Both EDTA‐plasma and serum, but mainly EDTA‐plasma, contained double‐positive EVs for CD9 and CD41a (Figure 3g and Figure S2), which confirmed the magnetic bead ELISA results obtained with Protocol 1 (Figure 2h,i). Furthermore, we measured the size of the particles with the nano flow cytometry, and the majority of the events were below 100 nm in size indicating that we are indeed analysing single EVs (Figure S2).

**FIGURE 4:**
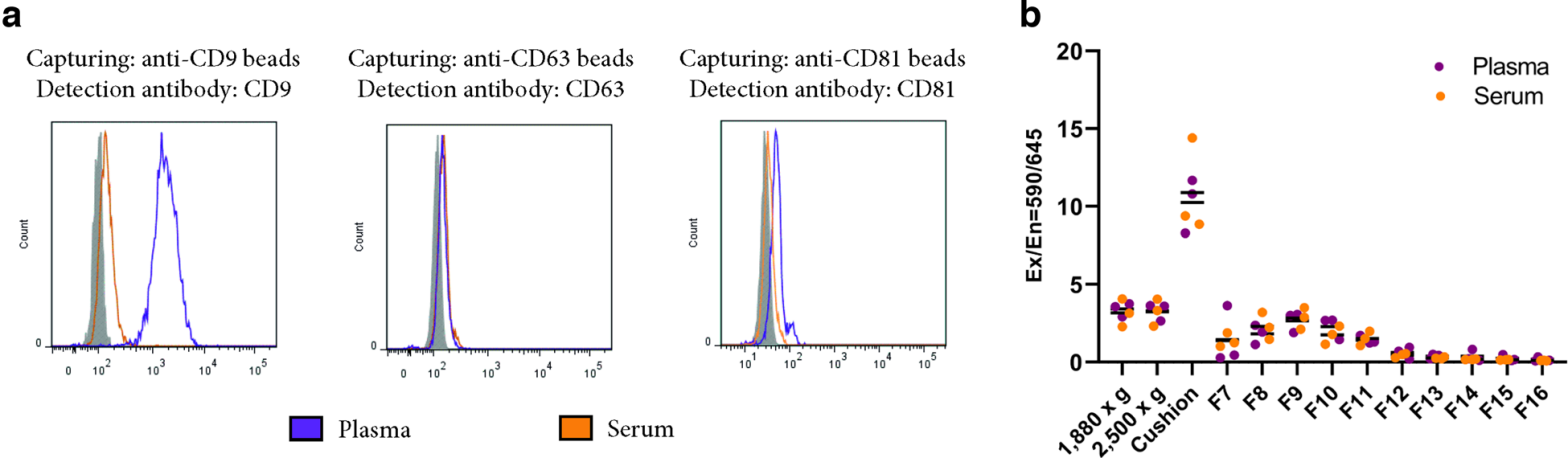
Serum clotting does not trap extracellular vesicles (EVs). (a) EVs were isolated with Protocol 2. Anti‐CD63, anti‐CD9 or anti‐CD81 beads were added directly to 1.2‐ml plasma and serum. Bead‐EV complexes were stained with anti‐CD63, anti‐CD9, anti‐CD63 or anti‐CD81, respectively, and analysed with flow cytometry. *N* = 1. b. mCherry‐CD63 EVs (15 μl) were spiked into blood collection tubes immediately after blood was drawn, and their presence was measured along all steps of Protocol 1 in both plasma and serum by measuring their fluorescence with a Varioskan™ LUX multimode microplate reader. *N* = 3

Additionally, Protocol 2, where anti‐CD9, anti‐CD63 and anti‐CD81 beads were added directly into EDTA‐plasma and serum, was also used to evaluate if certain EV subpopulations were lost during EV isolation with Protocol 1. By flow cytometry of the bead‐EV complexes, it was clear that the major difference between EDTA‐plasma and serum was the CD9^+^ EVs that were much more prominent in EDTA‐plasma than in serum, while little difference was seen for CD63^+^ and CD81^+^ EVs (Figure 4a and Table S1). The antibody clone used on the beads to capture EVs is proprietary by the company making the beads, and we could therefore not exclude that the clone used for capturing the EVs on the beads was the same as our detecting antibodies. We therefore next wanted to exclude that the capturing antibodies on anti‐CD63 and anti‐CD81 beads were competing with the binding sites for the detection antibodies and therefore gave rise to the lower signal for CD81 and CD63 compared to CD9. To test this, we used cell‐line‐derived EVs that we knew were positive for CD9, CD63 and CD81. From this control it was clear that CD63 and CD81 could be detected equally well as CD9, when all EV types were captured by beads with the same respective tetraspinin (Figure S3A). We, therefore, concluded that, because no EV isolation with SEC or density cushion/gradient had been applied for the plasma and serum samples, the difference in CD9^+^ EVs between EDTA‐plasma and serum was determined to be a biological difference and not a difference induced by technical issues during EV isolation or detection.

Furthermore, we next wanted to determine whether the increased presence of EV markers (CD9 and flotillin‐1) and platelet markers (CD41a and P‐selectin) in EDTA‐plasma compared to serum was due to these EVs being trapped in the clot produced in serum. To test this, we used cell line‐derived EVs expressing mCherry fused to CD63. Using these vesicles, we could avoid EV labelling protocols because it has been shown that these have several pitfalls (Simonsen, 2019; Simonsen & Kromann, 2021). The mCherry‐engineered EVs were spiked into the blood collection tube immediately after the blood had been drawn, and their presence along the isolation process of Protocol 1 was determined. No difference in mCherry was observed at any step during the EV‐isolation protocol between EDTA‐plasma and serum, suggesting that these spiked EVs were not trapped in the serum clot (Figure 4b).

All together, these results argue that EDTA‐plasma and serum EVs have similar densities, regardless of which EV marker is evaluated, and that our previous isolation methodology does not lose different types of EVs in serum. Furthermore, it is evident that EDTA‐plasma contains more platelet‐derived EVs than serum. Additionally, it is suggested that CD9^+^ vesicles are not trapped in the clot, and instead, the differences in CD9^+^ vesicles are likely to be at least partly a result of post‐collection events taking place in the EDTA tubes.

### 3.3 Few EVs in EDTA‐plasma and serum contain both CD81 and CD63

**FIGURE 5:**
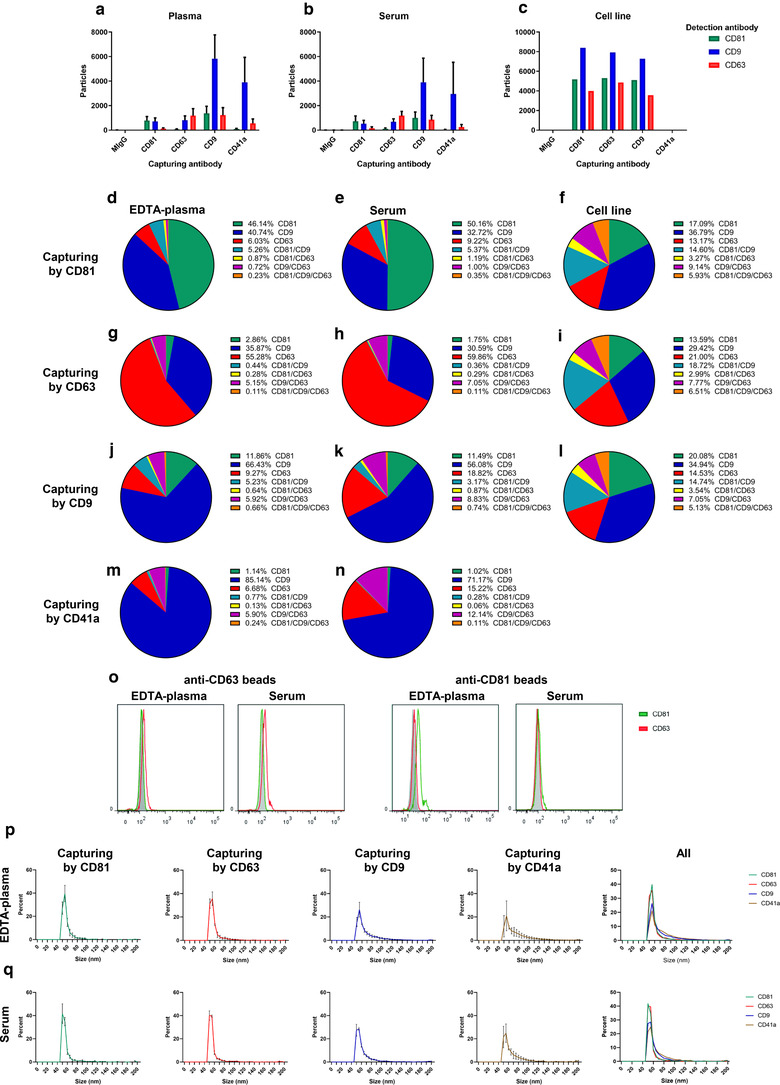
Few extracellular vesicles (EVs) in plasma and serum are double‐positive for CD63 and CD81. EVs were isolated with Protocol 1 for panels (a–n) and (p–q) (the starting volume of plasma and serum was 6 ml) and with Protocol 3 for panel O (the starting volume of plasma and serum was 1.2 ml). a–c. Thirty‐five microliters from fraction 9 were analysed with SP‐IRIS using the Tetraspanin Plasma kit on an ExoView™ R100 instrument. This kit captures EVs with anti‐CD81, anti‐CD63, anti‐CD9 and anti‐CD41a antibodies. On the chip is also the mouse IgG as a negative control. The anti‐CD81‐CF555, anti‐CD9‐CF488 and anti‐CD63‐CF647 antibodies were used for detection. *N* = 3 for plasma and serum. *N* = 1 for cell‐line‐derived EVs. Data are presented as the mean ± SEM. d–n. The data from panels A–C were analysed in depth to determine the percentage of single, double and triple‐positive (CD81, CD9, CD63) EVs captured with CD81, CD63, CD9 and CD41a. o. Anti‐CD63 and anti‐CD81 beads were added directly to the plasma and serum samples and were then analysed with flow cytometry to determine their expression of CD63 and CD81. Representative graphs are shown. *N* = 2. p,q. Size of EVs from plasma (p) and serum (q) captured by anti‐CD81, anti‐CD63, anti‐CD9 and anti‐CD41a as determined by the ExoView™ R100 instrument. *N* = 3 for plasma and serum. Data are presented as the mean ± SEM

To further determine the presence of CD9, CD63, CD81 and CD41a on EVs, we used SP‐IRIS. SP‐IRIS involves capturing EVs on a chip with anti‐CD9, anti‐CD63, anti‐CD81 and anti‐CD41a and then further staining for CD9, CD81 and CD63. We chose again to focus our analysis on one of the SEC fractions with the highest protein to particle ratios by analysing fraction 9 from both serum and EDTA‐plasma. EVs captured on the chip by the platelet protein CD41a were predominantly CD9^+^ (Figure 5a,b,m and n), thus supporting our findings from the magnetic bead ELISA (Figure 2h,i) and again suggesting that the increased presence of CD9^+^ EVs in EDTA‐plasma compared to serum is due to the release of EVs from platelets. The number of CD63^+^/CD41a^+^ EVs was higher in serum than in EDTA‐plasma (Figure 5m,n), also supporting our findings from the magnetic bead ELISA (Figure 2h,i). Interestingly, we also observed that CD81‐captured vesicles were only weakly positive for CD63 and vice versa, while both were positive for CD9 to a greater degree (Figure 5a,b,d,e,g and h). This suggests that very few CD63^+^/CD81^+^ double‐positive EVs are present in either EDTA‐plasma or serum, and this validates previous results for EVs isolated from serum (Bachurski et al., 2019; Mizenko et al., 2021). When we analysed the supernatant from a human mast cell line, we did not observe any such difference, and instead triple tetraspanin‐positive EVs were detected (Figure 5c,f,i and l). CD9‐captured vesicles in EDTA‐plasma and serum were positive for all three tetraspanins although they were mainly positive for CD9 (Figure 5a,b,j and k). Thus, the majority of EVs in serum and plasma are positive for CD9, which is in line with the magnetic bead ELISA, nanoflow cytometry and Western blot data (Figs. 2d, 2f **–** i and 3e **–** g). To further validate the non‐mutual existence of CD63 and CD81 on the same vesicles observed with SP‐IRIS, we used Protocol 2 again (Protocol 2 in Figure 1) and isolated EVs by adding anti‐CD63 and anti‐CD81 beads directly to the EDTA‐plasma and serum samples and then stained the bead‐captured EVs with anti‐CD63 and anti‐CD81. Also with this method, it was clear that CD63‐captured EVs expressed very little CD81 and vice versa (Figure 5o and Table S2). To eliminate the possibility that steric hindrance was the reason we could not detect CD63 on CD81‐captured EVs and vice versa, we again used the cell line‐derived EVs. It was then clear that CD63 could be detected on anti‐CD81 beads and vice versa (Figure S3B). With this method, we could therefore validate that there was no detectable signal for CD63 in EDTA‐plasma and serum when vesicles were captured with anti‐CD81 beads and vice versa (Figure 5o).

Next, we utilized the possibility to obtain the size of the EVs captured by SP‐IRIS. Although all EVs showed similar sizes of around 50–70 nm, it was evident that the EVs captured by anti‐CD41a and anti‐CD9 had a tail of larger EVs as well (Figure 5p,q). This small size also suggests that single EVs had indeed been captured, rather than aggregates of EVs, thus giving rise to the double‐positive signals observed for the different tetraspanins and CD41a.

Together, these results support the conclusion that CD9 and CD41a are present on the same EVs. Furthermore, they argue that CD63 and CD81 are mainly present on separate EV subpopulations in EDTA‐plasma and serum; however, both subpopulations are seemingly positive for CD9. It should be noted, however, that we cannot exclude that there is also a subpopulation that expresses all three tetraspanins, although the SP‐IRIS data suggest that this population is very rare (Figure 5d **–** n).

### 3.4 The distribution of different tetraspanins among subpopulations of EVs in EDTA‐plasma

To further characterise the CD63^+^/CD81^−^, CD63^−^/CD81^+^ and CD9^+^/CD41a^+^ subpopulations of EVs, we next used Protocol 2 again to isolate EVs by adding anti‐CD9, anti‐CD63 and anti‐CD81 beads (Figure 1) directly to the EDTA‐plasma. We sought to analyse the subpopulations captured by the beads by eluting them off the beads for further analysis. Again, we used cell culture EVs to evaluate the elution buffers because large quantities of EVs were needed during the protocol optimisation. However, none of the three buffers evaluated to remove intact EVs had any effect when tested on cell‐derived EVs (Figure S4A). The only efficient elution buffer was 2% SDS (Figure S4A), which still allowed for further analysis with Western blot and mass spectrometry, but not single EV analysis because the EVs were lysed. Western blot showed that flotillin‐1^+^ and CD9^+^ cell line‐derived EVs could be eluted from all three types of beads (Figure S4B). As there was no reason to believe that these buffers would behave differently on EDTA‐plasma EVs, we decided to continue with the 2% SDS buffer with the EDTA‐plasma. Importantly, we could validate that EVs isolated from EDTA‐plasma could also be eluted from the beads with 2% SDS (Figure S4C).

**FIGURE 6:**
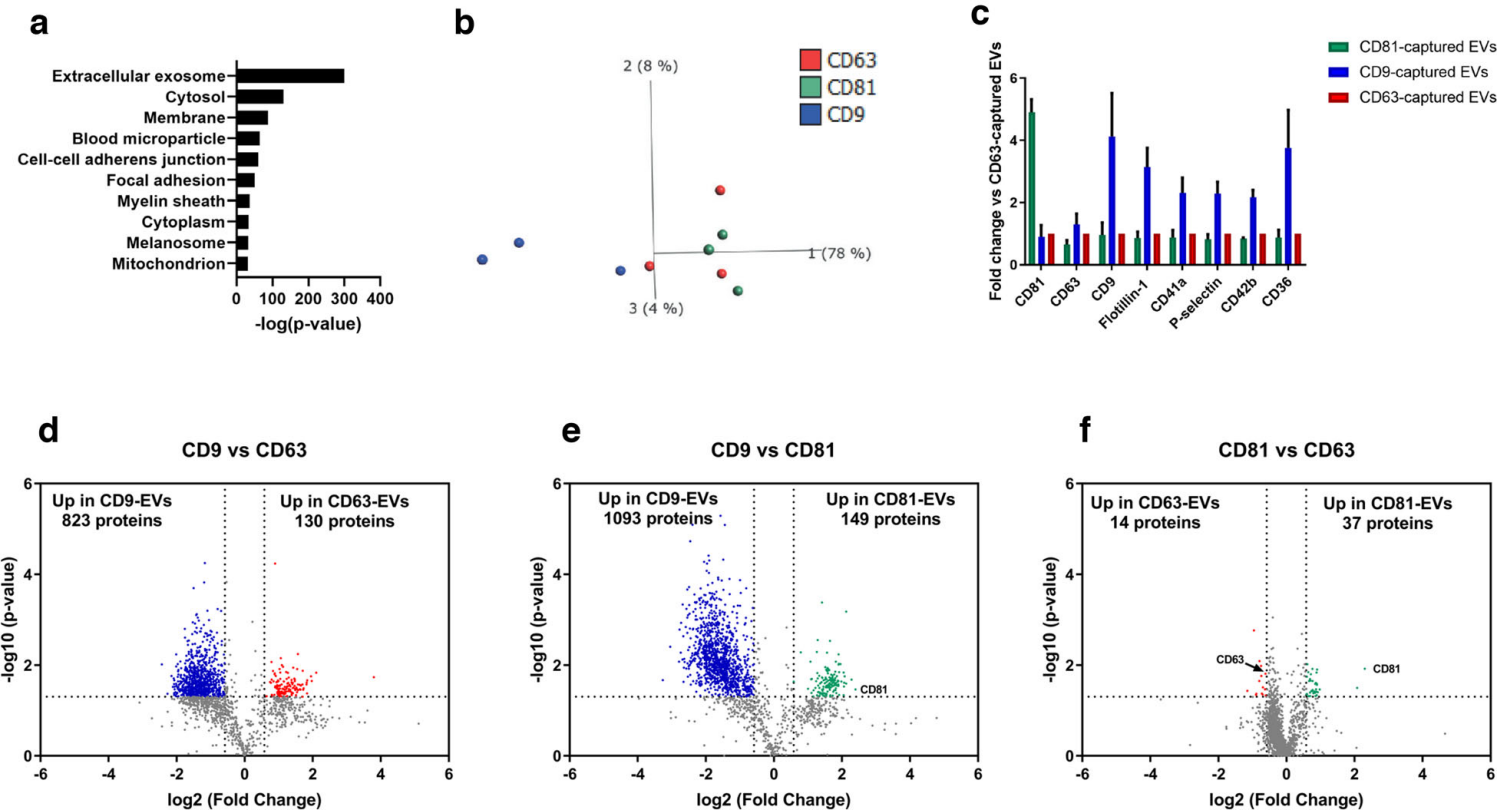
Distribution of different tetraspanins among subpopulations of extracellular vesicles (EVs) in plasma and serum. EVs were isolated with Protocol 2. EVs were eluted off the anti‐CD9, anti‐CD63 and anti‐CD81 beads from plasma with 2% SDS, and 40 μg per samples was quantified with tandem mass tag LC‐MS/MS. *N* = 3. a. The top 10 ‘cellular compartments’ GO terms associated with the proteome of plasma EVs. b. PCA illustrating the relationship between the proteome of CD9‐captured EVs (blue), CD63‐captured EVs (red) and CD81‐captured EVs (green). c. Fold change of the three tetraspanins, flotillin‐1 and four platelet proteins. The fold change was calculated compared to the CD63‐captured EVs. d–f. Volcano plots of the proteome of CD9 versus CD63 (f), CD9 versus CD81 (g), and CD81 versus CD63 (h). Dotted lines indicate cut‐offs, which were 1.3 on the *Y*‐axis (corresponding to *p* <  0.05) and 0.67 on the *X*‐axis (corresponding to fold change >1.5)

To gain further insight into the different tetraspanin subpopulations of EVs, quantitative mass spectrometry was used on EVs isolated from EDTA‐plasma with anti‐CD9, anti‐CD81 and anti‐CD63 beads. We focused on EDTA‐plasma because it contained more of the CD41a^+^ EV subpopulation and provided more material in general. A total of 2359 proteins were identified, with 1784 proteins also being quantified with tandem mass tag (TMT) analysis. The cellular components‐related GO terms most associated with the proteins identified in the EDTA‐plasma EVs were ‘extracellular exosome’, ‘cytosol’, ‘membrane’, and ‘blood microparticle’ (Figure 6a). To visualise the relationship between the different tetraspanin subpopulations of EVs, a principle component analysis (PCA) was performed including all quantified proteins. Component 1, representing the majority (78%) of the variability distinguished the CD9‐captured vesicles from the CD63‐captured and CD81‐captured vesicle (Figure 6b). Importantly, CD81 and CD9 were enriched in the CD81‐ and CD9‐captured vesicles, respectively (Figure 6c). However, CD63 was most enriched in the CD9‐captured vesicles (Figure 6c). Flotillin‐1 together with the platelet markers CD41a, p‐selectin, CD42b and CD36 were all enriched in the CD9‐captured vesicles compared to the other two subpopulations (Figure 6c). When all the tetraspanin subpopulations were compared to each other, it was clear that the CD9‐captured EVs were the most dominant and most distinct population (Figure 6d **–** f). In total, 823 and 1093 proteins were upregulated in the CD9‐captured vesicles compared to CD63‐ and CD81‐captured vesicles, respectively (Figure 6d,e). On the other hand, only 130 and 149 proteins were upregulated in CD63‐ and CD81‐captured vesicles, respectively, compared to CD9‐captured vesicles (Figure 6d,e). Surprisingly, only 14 and 37 proteins were upregulated in CD63‐ and CD81‐captured vesicles, respectively, when compared to each other (Figure 6f).

Together, these results argue that CD9‐EVs are the most common EVs in EDTA‐plasma. While CD81 and CD9 were highly enriched in the EVs isolated with these particular antibody‐coated beads, this was not observed for CD63, which suggests that CD63 is present in multiple subpopulations of EVs. Although the SP‐IRIS and flow cytometry data (Figure 5a **–** o) suggest that CD63 and CD81 are rarley present on the same EVs, the LC‐MS/MS data showed little difference in other protein components between these two subpopulations of EVs.

### 3.5 Fewer residual platelets are present in serum and ACD‐plasma compared to EDTA‐plasma and two centrifugations of plasma is crucial to reduce platelets

**FIGURE 7:**
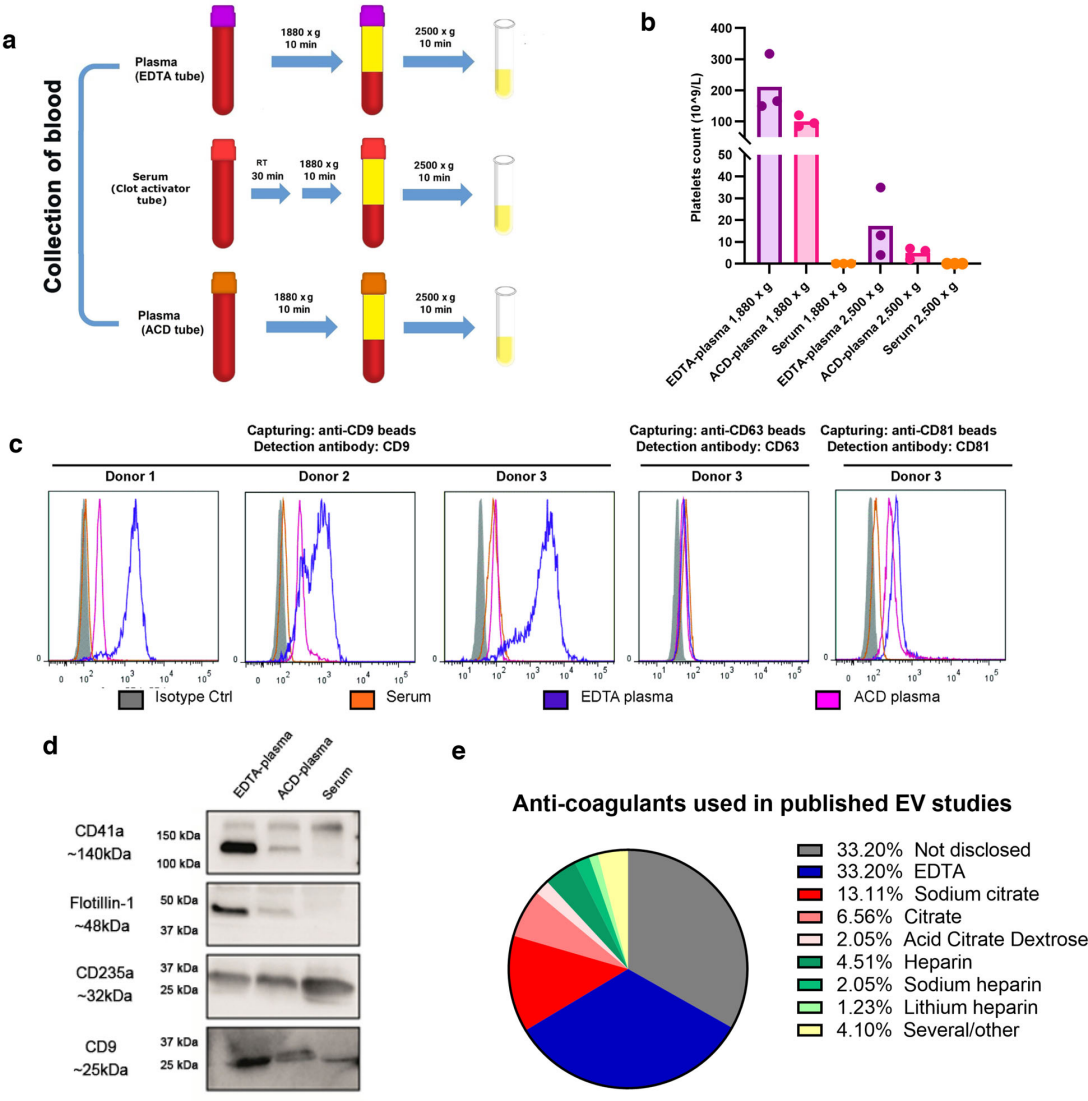
EDTA‐plasma contains more residual platelets than ACD‐plasma and serum. a. ETDA‐plasma, ACD‐plasma and serum were collected according to the schematic overview. b. Number of residual platelets in EDTA‐plasma, ACD‐plasma and serum as measured by a Sysmex automated counter. *N* = 3. c. Anti‐CD63, anti‐CD9 or anti‐CD81 beads were added directly to EDTA‐plasma, ACD‐plasma and serum. Bead‐EV complexes were stained with anti‐CD63, anti‐CD9 or anti‐CD81, respectively, and analysed by flow cytometry. *N* = 3 for anti‐CD9 beads and *N* = 1 for anti‐CD63 and anti‐CD81 beads. d. EVs were eluted off the anti‐CD9 beads from EDTA‐plasma, ACD‐A plasma and serum with 2% SDS, and the presence of CD41a, flotillin‐1, CD235a and CD9 was determined by Western blot. In total, 36 μg/well was loaded. e. The EV‐TRACK knowledgebase was searched for all human plasma studies, and the methods sections of the identified studies were manually read to identify which type of anti‐coagulant had been used. In total, 244 studies were analysed

Because cells are trapped in the clot formed during the serum collection protocol, plasma can contain more residual platelets than serum. Some of the techniques used in this project, such as ExoView and nanoFCM, measure the size of the objects that are positive for CD41a, and we could conclude that these objects are of the same size as EVs and therefore are likely to be platelet‐derived EVs. However, for other techniques such as Western blot and mass spectrometry, we do not know the sizes of the objects that are positive for CD41a. Therefore we can not exclude that some part of the CD41a signal may actually be from residual platelets or platelet fragments themselves contaminating the isolated EVs. We therefore next decided to determine the presence of platelets and the possible risk of contamination of residual platelets in the EV isolates. It has previously been suggested that ACD‐A inhibits platelet activation and results in the most stable levels of microparticles in plasma compared to other anticoagulants (Gyorgy et al., 2014). Therefore, we next also included ACD‐A plasma collection tubes and from each subject blood was collected into serum, EDTA‐plasma and ACD‐plasma tubes so that all samples were paired (Figure 7a). First, we counted the residual platelets in EDTA‐plasma, ACD‐plasma and serum. No platelets (or below the detection limit) were found in serum after both the 1880 × *g* and 2500 × *g* centrifugations (Figure 7b). In EDTA and ACD‐plasma, an 89%–98% reduction in platelets was first observed after the second centrifugation. Both EDTA‐ and ACD‐plasmas still contained significantly more residual platelets than serum after the second centrifugation (Figure 7b).

Next, we utilised Protocol 2 and flow cytometry to evaluate the presence of CD63, CD81 and CD9 in plasma and serum now also including ACD plasma. It was shown that ACD‐plasma contained fewer CD9^+^ EVs than EDTA‐plasma, but more or similar amounts as serum (Figure 7c and Table S3). Similar trends were seen for CD81^+^ EVs, while no difference was seen for CD63^+^ EVs. When the EVs were eluted off the beads and analysed with Western blot, CD41a, flotillin‐1 and CD9 were the most expressed in EDTA‐plasma‐derived EVs and were expressed less in ACD‐plasma and serum‐derived EVs (Figure 7d). CD235a showed similar expression in all of the samples.

Because we could observe a difference in the presence of residual platelets and tetraspanin expression on EVs in EDTA‐plasma, and ACD‐plasma we were interested in which anti‐coagulants are commonly used in EV studies. We, therefore, used EV‐Track to identify plasma EV papers to further evaluate their methods sections. Thirty‐three percent of the EV studies had used EDTA, 22% one of the citrates and 8% one of the heparins (Figure 7e). Most concerning was that 33% of the studies did not disclose which anti‐coagulant they had used.

**FIGURE 8:**
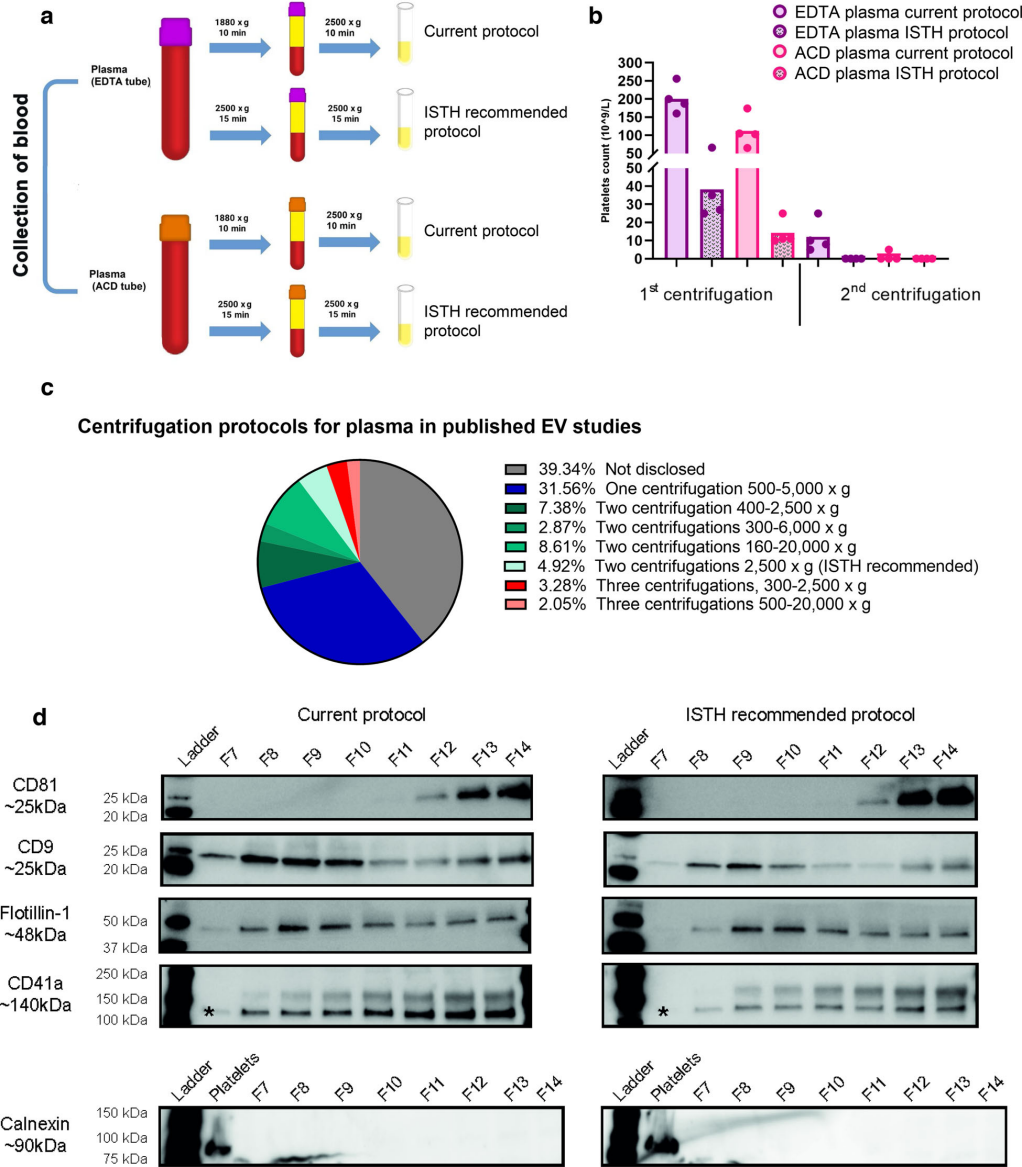
Two centrifugations are crucial to reduce residual platelets. a. ETDA‐ and ACD‐plasmas were collected according to the schematic overview, where our current protocol was compared to the one recommended by the ISTH. b. The number of residual platelets in EDTA‐ and ACD‐plasmas after the two isolation protocols was as measured by a Sysmex automated counter. *N* = 4. c. The EV‐TRACK knowledgebase was searched for all human plasma studies, and the methods sections of the identified studies were manually read to identify how the blood/plasma was centrifuged prior to freezing and EV isolation. In total, 244 studies were analysed. d. EVs were isolated with Protocol 1 starting with 6‐ml plasma. The presence of the EV markers CD81, CD9, and flotillin‐1, the platelet marker CD41a and the endoplasmic reticulum marker calnexin was determined in SEC fractions 7–14 (36 μl/fraction) by Western blotting. CD41a is detected at 140 kDa, which is the lower band highlighted with *. Platelets pelleted from platelet‐rich‐plasma were used as a positive control for calnexin

The ISTH has recommended that two centrifugations of 2500 × *g* for 15 min should be used to obtain platelet‐poor plasma (Coumans et al., 2017). Although our protocol contains two centrifugations and the last one is 2500 × *g*, we wanted to evaluate if the difference in speed of the first centrifugation and the difference in time of the second centrifugation made an impact on the number of residual platelets. Because serum did not contain any platelets with our current centrifugation method, we focused on ACD‐ and EDTA‐plasmas. ACD‐ and EDTA‐plasmas were collected from the same person, and half of each sample was used for our current plasma isolation method and the other half for the protocol recommended by the ISTH, resulting in all samples being paired (Figure 8a).

In EDTA‐ and ACD‐plasmas, an 82%–87% reduction in platelets was observed during the first centrifugation when the ISTH method was compared to our current method (Figure 8b). After the second centrifugation, both the EDTA‐ and ACD‐plasmas from the ISTH method contained no platelets (or below the detection limit). For our current method, 3 out of 4 ACD‐plasma samples contained no platelets (or below the detection limit), while all EDTA‐plasma samples contained residual platelets.

Because the observed difference in the presence of residual platelets between one and two centrifugations as well as between different g forces and centrifugation times we were interested in what protocols are commonly used in EV studies to generate plasma from blood. We, therefore, used the same plasma studies previously identified in EV‐Track to further evaluate their methods sections. Thirty‐two percent of the EV studies had only used one centrifugation, 24% had used some version of two centrifugations and 5% had used some version of three centrifugations (Figure 8c). Most concerning was that almost 40% of the studies did not disclose how they isolated plasma from blood.

Lastly, we wanted to determine how our EV isolates were affected by residual platelets. We therefore used EDTA‐plasma and divided it in two to use half for our current protocol for plasma preparation and the other half for the ISTH protocol to prepare plasma. We then used Protocol 1 to isolate EVs. The Western blot of the SEC fractions showed that CD9 and CD41a were detected using both plasma isolation protocols although there was a trend for a slightly stronger signal with our current protocol (Figure 8d). Flotillin‐1 did not show a prominent difference between the two plasma isolation protocols, while CD81 had a tendency to be stronger in the ISTH protocol. Calnexin was not detected in any of the SEC fractions with either protocol (Figure 8d).

Together, this suggests that EDTA‐plasma contains more residual platelets and CD9^+^ EVs than ACD‐plasma and serum and the differences in CD9^+^ vesicles might therefore be at least partly due to post‐collection activation of platelets in EDTA tubes. Although residual platelets may also partly contribute to some of the CD9 and CD41 signals seen in the EDTA‐plasma samples, the lack of Calnexin in all fractions suggests little contamination by platelets or other cells. CD63^+^ and CD81^+^ EVs, in contrast, were less affected by the type of anticoagulant used. Furthermore, two centrifugations of plasma appear to be crucial to reduce platelets and preferably two times at 2500 × *g* for 15 min. However, less than 5% of the plasma‐EV studies we evaluated used this protocol.

## 4 DISCUSSION

We have previously described the importance of both density isolation and SEC to separate EVs in plasma from other components in blood, including lipoproteins (Karimi et al., 2018). Here we extended these findings by comparing EV phenotypes in EDTA‐plasma, ACD‐plasma and serum using NTA, Western blot, SP‐IRIS, conventional and nanoflow cytometry, magnetic bead ELISA and proteomics. Through this detailed approach, we could identify multiple subpopulations of EVs in both EDTA‐plasma and serum. Importantly, EDTA‐plasma contained more CD9^+^/CD41a^+^ double‐positive EVs than serum, suggesting a platelet origin for this subpopulation. Spike‐in experiments suggested that EVs were not trapped in the serum clot, and therefore the increased amount of CD9^+^ EVs in EDTA‐plasma was not due to loss of EVs present in serum. By comparing different anticoagulants, we instead suggest that the CD9^+^/CD41a^+^ EVs are released during blood collection or are released in vitro in the collection tube. Furthermore, few EVs were positive for both CD63 and CD81, suggesting that several different tetraspanin‐positive subpopulations of EVs are present in plasma and serum. Additionally, we determined the number of residual platelets and their effects on the EVs that were isolated. EDTA‐plasma contained more residual platelets than ACD‐plasma and serum, and two centrifugation steps during the plasma isolation were crucial to minimize the number of residual platelets prior to EV isolation.

First, we demonstrated that the majority of the EVs present in both plasma and serum were CD9^+^, and furthermore that these CD9^+^ EVs were considerably enriched in EDTA‐plasma compared to ACD‐plasma and serum. Importantly, we found that the platelet marker CD41a was present on the same individual EVs as CD9. Second, our results suggest that very few CD63^+^/CD81^+^ double‐positive EVs are present in both EDTA‐plasma and serum and that CD63^+^ EVs vesicles are enriched in serum. Interestingly, CD63 was most prominent in SEC fraction 10 according to Western blot, while the CD81 signal was the highest in fraction 14, which further implies that these tetraspanins are present in different EVs, with CD81 being associated with smaller‐sized EVs. However, we cannot exclude that for some of the techniques, such as the bead‐based flow cytometry, the expression of CD63 and CD81 is below the detection limit or that aggregates have bound to beads in bead ELISA giving rise to some of the CD9/CD41a double positivity. However, the SP‐IRIS analysis required much fewer EVs and both CD63 and CD81 were detected on the CD9‐captured EVs, suggesting that we would be able to detect CD63 on CD81 EVs and vice versa if they were truly there. SP‐IRIS was also used to evaluate the number of double‐positive particles for CD41a and CD9, and the size distribution showed that these particles were 50–70 nm, which argues against these being aggregates. Importantly, all three of our isolation methods are none‐pelleting methods, so induced aggregation during EV isolation should be limited. Another potential technical problem could be steric hindrance preventing the antibodies from binding to CD63 and CD81 on the same EV. However, this was not a problem for the cell line‐derived EVs where CD63‐captured EVs were positive for CD81 and vice versa. Furthermore, we previously observed with SP‐IRIS that both large and small EVs from human tumour tissue are double‐positive for CD63 and CD81 (Crescitelli et al., 2020), suggesting that there is no steric hindrance to identifying both CD63 and CD81 on the same EV, and we have no reason to believe that it would be different for serum and plasma EVs. Additionally, two previous studies have observed similar results as us for CD81 and CD63 on EVs in serum (Bachurski et al., 2019; Mizenko et al., 2021). Importantly, we are not suggesting that a CD63^+^/CD81^+^ subpopulation does not exist at all in serum and plasma, but in our hands, this subpopulation is very rare.

It has been suggested that each platelet can carry up to 50,000 CD9 molecules (Protty et al., 2009). This makes CD9 one of the most common molecules on the platelet cell surface, together with the CD41/CD61 receptor (also called integrin αIIb and integrin β3) with around 80,000 copies per platelet (Wagner et al., 1996). Furthermore, CD9 co‐localises with CD41/CD61 on the platelet surface and on the inner surface of α‐granule membranes (Brisson et al., 1997; Longhurst et al., 1999). Additionally, it has been shown that CD9 and CD63 are present in distinct structures in human platelets (Brisson et al., 1997) as well as in mouse breast cancer cells (Bobrie et al., 2012) and HeLa cells (Mathieu et al., 2021). It has also been shown that CD9 is primarily located in the plasma membrane while CD63 is located in intracellular compartments, primarily endosomes (Mathieu et al., 2021; Tkach et al., 2018; Verweij et al., 2018). CD63 is also localised in the membranes of α‐granules and dense granules in resting platelets. Upon stimulation and granule exocytosis, CD63 is expressed on the plasma membrane and can co‐localise with CD9 (Komatsuya et al., 2020).

Regarding the location of CD81, it has been reported to co‐localise with CD63 in the endosomes (Verweij et al., 2018) and with CD9 in the plasma membrane (Fordjour et al., 2019; Kowal et al., 2016) in cell lines. Moreover, it has been suggested that platelets do not express CD81 (Tomlinson, 2009), and in‐depth analysis of platelet‐derived EVs has shown that they have no detectable CD81 (Koliha et al., 2016; Sung et al., 2019). In contrast, CD81 is highly expressed on B, T and NK lymphocytes, and weakly or moderately on myeloid cells, but it is not expressed on erythroid cells (Ma et al., 2001). It is possible that additional subpopulations of EVs may exist in the circulation that have not been identified here; however, our data argue for the presence of the following subpopulations of EVs in EDTA‐plasma and serum. First, EVs released by platelets, thus carrying CD41a, were also CD9^+^ and carried p‐selectin and flotillin‐1. A less prominent CD41a^+^/CD9^+^/CD63^+^ subpopulation was also observed, as it was clear that not all CD41a^+^/CD9^+^ EVs had detectible levels of CD63 on their surface. Additionally, a less abundant subpopulation of CD41a^+^/CD63^+^ EVs without CD9 expression may also exist. The data further suggest the presence of a CD63^+^ population that is not released by platelets, because it does not carry CD41a. When it comes to CD81^+^ EVs, they most often also express CD9 but are most likely not released by platelets because they do not express CD41a and because CD81 is not found in platelets. In general, CD81 is the least‐expressed tetraspanin on circulating EVs. Because SP‐IRIS showed that capturing by the three tetraspanins generated signals for all three tetraspanins, although low numbers for CD63/CD81 were observed, we cannot exclude that there is also a small population positive for all three tetraspanins (CD9^+^/CD63^+^/CD81^+^), but in our hands, this subpopulation is very rare.

Together, these data argue that the CD9^+^/CD41a^+^ double‐positive EVs identified in EDTA‐plasma and serum in our study are platelet‐derived, while CD81^+^ EVs are most likely released neither by platelets nor erythrocytes. The CD63^+^ EVs might still be released by platelets, but although CD63 might be enriched in endosomes, its location is dynamic and we cannot conclude which compartment these EVs originate from, while the CD9^+^/CD41a^+^ double‐positive EVs probably originate from the plasma membrane. This may suggest that mainly CD81 and perhaps also CD63 are more reliable tetraspanins to evaluate in plasma and serum if one is interested in non‐platelet EVs, while CD9 probably is a better marker if one is focusing on platelet‐derived EVs. CD9 is also the tetraspanin most affected by the choice of anti‐coagulants and may therefore be troublesome to compare between different studies.

To further understand whether the EDTA‐plasma and serum EVs were small or large, we determined the presence of mitofilin and ADAM10 because they have previously been shown to distinguish large and small EVs, respectively (Crescitelli et al., 2020; Kowal et al., 2016). Mitofilin was only present in the EDTA‐plasma sample, which suggests that at least some part of the platelet‐derived vesicles released in the tube after collection were large EVs. ADAM10 was detected in both EDTA‐plasma and serum EVs; however, the band was stronger in EDTA‐plasma. This suggests that small EVs are present in both EDTA‐plasma and serum, but it also implies that small EVs are, at least to some degree, released in the tube after collection. Interestingly, CD63 was one of the few markers that were enriched in serum. While both CD63 and CD42 (also known as glycoprotein Ib and which is expressed on platelets) have been found on both small and large EVs from TRAP‐activated platelets, CD63 is enriched in the small EVs while CD42 is enriched in large EVs (Heijnen et al., 1999). Additionally, a cryo‐electron microscopy study showed that CD41^−^ EVs were mainly EVs smaller than 300 nm (Brisson et al., 2017). Furthermore, two types of CD63^+^ EVs were released from the platelets – one population that was >200 nm with weak CD63 expression, and another population that was <200 nm with strong CD63 expression (Brisson et al., 2017). These electron microscopy studies suggested that smaller EVs are CD63^+^, while larger EVs are CD41^+^ and CD42^+^. We currently do not know the size of our CD63^+^ subpopulations of EVs and we cannot exclude that the EVs released by activated platelets in the studies highlighted here are different compared to the EVs present in the circulation, but this could suggest that the CD63^+^ subpopulation is smaller than the CD9^+^/CD41a^+^ subpopulation. Our SP‐IRIS data may support this because the CD9^+^ and CD41a^+^ EVs had a trend of being larger than CD81^+^ and CD63^+^ vesicles. However, it is important to note that the shift was small and this instrument only measures the size of EVs between 50 and 200 nm, so we cannot make a conclusion for EVs larger than 200 nm with this technique.

Many researchers have proposed that the activation of the clotting cascade could lead to artificial platelet activation and vesiculation during blood collection, which would result in the release of platelet‐derived EVs. If this is the case, then there would be an increased presence of platelet‐derived EVs in serum compared to plasma. However, few studies have actually evaluated whether this is true or not. A recent study showed that more CD61^+^ and CD41^+^ EVs were present in serum compared to plasma collected with ACD‐A, citrate or EDTA tubes (Palviainen et al., 2020), while another study showed no difference in CD41 in EVs isolated from serum and heparin‐tube‐collected plasma (Muller et al., 2014). In the current study, we observed more CD41a^+^ EVs in EDTA and ACD‐collected plasma compared to serum using multiple methods for EV analysis. We currently do not know what is behind this discrepancy in the results. However, there are several pre‐analytical factors that could explain the differences, including choice of anticoagulant, venipuncturing and handling of the collected blood, time of blood collection and fasting, the processing of the blood to generate plasma and serum, haemolysis and the removal of platelets (Baek et al., 2016; Clayton et al., 2019; Witwer et al., 2013). Further, the method of EV isolation such as the utilisation of ultracentrifugation, SEC or density gradients might also affect the results because different methods could enrich different subpopulations of EVs or could, in theory, preferentially damage some EV subpopulations. Lastly, the choice of method for characterisation can matter because the detection limits can be dissimilar, and in the current study, we, therefore, used multiple analytical methods to verify our results.

To evaluate if vesicles became trapped in the clot and if this could be the reason for fewer EVs being present in serum compared to EDTA‐plasma, we also spiked cell line‐derived EVs expressing mCherry fused to CD63 in the blood tubes prior to collecting EDTA‐plasma and serum. The reason for using cell‐line‐derived EVs from transfected cells over platelet‐derived EVs was that these vesicles did not have to be labelled and therefore the risk for micelles or unbound dye creating artefacts effecting the experiment was minimized. We could also avoid the risk of the dye spilling over to other membranes such as the lipoproteins particles after the labelling, which would also effect the results (Simonsen, 2019; Simonsen & Kromann, 2021). Our results showed no difference in the presence of mCherry‐EVs at any step during the EV‐isolation protocol between EDTA‐plasma and serum, suggesting that EVs are not trapped in the serum clot. However, we cannot exclude that platelet‐derived EVs might behave differently and at least partly be trapped in the clot. We clearly observed fewer CD9^+^/CD41a^+^ double‐positive vesicles in ACD‐plasma compared to EDTA‐plasma, which may suggest different degrees of platelet activation for the two anticoagulants. In a study where in vitro platelet activation was studied by monitoring the release of thrombospondin and platelet factor 4, it was shown that these were released in higher quantities after collection in heparin‐plasma or EDTA‐plasma compared to citrate; citrate, theophylline, adenosine and dipyridamole (CTAD); and ACD‐A plasma. Even when platelet activation was induced with thrombin, the levels in CTAD and ACD‐A plasma were lower than unstimulated citrate, heparin and EDTA‐plasma (Mussbacher et al., 2017). Additionally, the levels of microparticles (MPs) have been shown to be higher in EDTA‐ and heparin‐plasmas compared to citrate‐ and CTAD‐plasmas (Lacroix et al., 2012). Furthermore, in a study comparing the levels of platelet and non‐platelet‐derived MPs in plasma collected with citrate, ACD‐A, CPDA, CTAD and heparin tubes, it was shown that platelet inhibition in the ACD‐A tubes led to more stable in vivo MP levels, as well as less artificially induced MP release after in vitro stimulation by agitation and incubation at 37˚C compared to the other tubes (Gyorgy et al., 2014). Unfortunately, EDTA tubes were not included in that study because EDTA was not compatible with the flow cytometry analysis that was performed. Furthermore, one study showed that CD42b^+^ and CD9^+^ double‐positive EVs were enriched in EDTA‐plasma compared to heparin‐ and CPDA‐plasmas (Makhro et al., 2016). Together, these results suggest that platelets are less activated in ACD‐A tubes. Additionally, we observed a difference in residual platelets depending on the anti‐coagulant used. While serum had no residual platelets (or below detection limit), both EDTA‐ and ACD‐plasmas had residual platelets after 1880 × *g* for 10 min followed by 2500 × *g* for 10 min with a trend of ACD‐plasma having less platelets and less variation between subjects than EDTA‐plasma. Additionally, we compared our plasma isolation protocol (1880 × *g* for 10 min followed by 2500 × *g* for 10 min) with the one recommended by the ISTH (2500 × *g* for 15 min followed 2500 × *g* for 15 min) to determine how the length and *g* force of the centrifugations affected the number of residual platelets. A larger difference in residual platelets was observed for EDTA‐plasma compared to ACD‐plasma between the two plasma isolation protocols. Importantly, we did not observe a major difference for CD41a, flotillin‐1, CD9 or CD81 in our EV isolates from EDTA‐plasma when we compared the two plasma isolation protocols. Additionally, Calnexin was not identified in any of the SEC fractions. First, this suggests that the CD9, CD41a and flotillin‐1 signal was not contributed by residual platelets but instead by platelet‐derived EVs. However, we cannot completely exclude that some residual platelets were still present in the plasma just under the detection limit of the Symax instrument. However, the lack of Calnexin also suggests that no residual platelets or other cells were present. Second, the CD81 expression indicated that EVs were not lost when the stronger and longer centrifugations were applied during plasma isolation. However, it is of concern that many EV studies do not report which anti‐coagulant and/or plasma isolation protocol that has been used or are only ultracentrifuging the blood samples ones to obtain plasma. One previous study suggested that one centrifugation at 5000 × *g* for 20 min could be equally efficient at reducing the numbers of residual platelets as the ISTH protocol (Rikkert et al., 2020). However, none of the EV studies reporting one centrifugation in our literature search centrifuged at 5000 × *g* for 20 min, and all protocols were shorter or more importantly had a lower *g* force. Because residual platelets can affect downstream analysis, the importance of evaluating them in EV studies has been highlighted previously (Clayton et al., 2019). However, few EV studies have performed this quality control, which should be important to perform in future research (Palviainen et al., 2020; Rikkert et al., 2020). This is especially because different methods for isolating EVs have different abilities to separate the residual platelets from the EVs prior to EV analysis. It is also important to highlight that if platelets have not been properly removed prior to freezing of plasma for EV‐isolation at a later time point, the platelets will be damaged during the freezing/thawing, and this will lead to increasing numbers of vesicles‐like particles that might contaminate the EV isolates. Depending on the aim of the study and the choice of EV isolation method and downstream analytical methods, different types of anticoagulants can be used for the collection of plasma, but two centrifugations should always be applied and reported.

In conclusion, this is the first study to identify seemingly distinct subpopulations of EVs in human plasma and serum using multiple isolation methods as well as several analytical methods. It is clear that platelet‐derived EVs can be identified in both plasma and serum and that the relative presence of the tetraspanins CD9, CD63 and CD81 is different in different subpopulations. We have previously described a rare subpopulation of EVs in human blood carrying the mitochondrial inner membrane molecules MTCO2 and Cox6c (Jang et al., 2019), and it is most likely that additional subpopulations of EVs exist both in healthy states and under pathological conditions. Our current study has focused on blood samples from healthy subjects, but the results have broad implications for future EV‐based biomarker discovery studies.

## CONFLICT OF INTEREST

J.L. and C.L. have developed multiple EV‐associated patents for putative clinical utilization. J.L. owns equity in Codiak BioSciences Inc. and Exocure Biosciences Inc. and consults in the field of EVs through Vesiclebio AB. C.L. owns equity in Exocure Bioscience Inc.

## Supporting information

SUPPORTING INFORMATION

SUPPORTING INFORMATION

SUPPORTING INFORMATION

SUPPORTING INFORMATION

SUPPORTING INFORMATION

SUPPORTING INFORMATION

SUPPORTING INFORMATION
