# Supplementary figures and images for: Tetraspanins distinguish separate extracellular vesicle subpopulations in human serum and plasma – Contributions of platelet extracellular vesicles in plasma samples
Source: J Extracell Vesicles. 2022 May 6;11(5):e12213. doi: 10.1002/jev2.12213 (PMC9077141; doi:10.1002/jev2.12213)

# Supplementary Figure 1

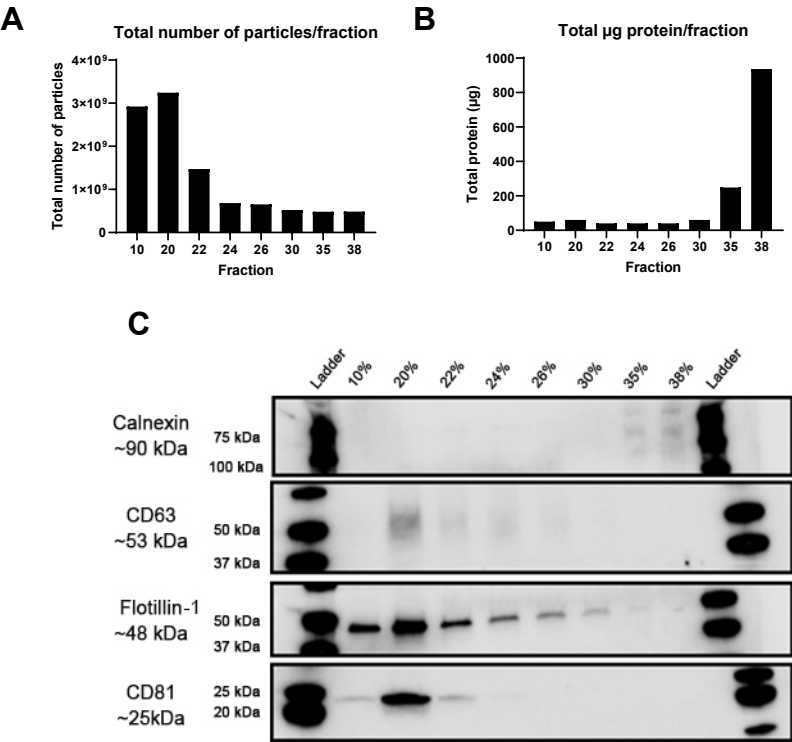

Supplement: Supplementary file 1 — SUPPORTING INFORMATION [file JEV2-11-e12213-s003.pdf]

# Supplementary Figure 2

A

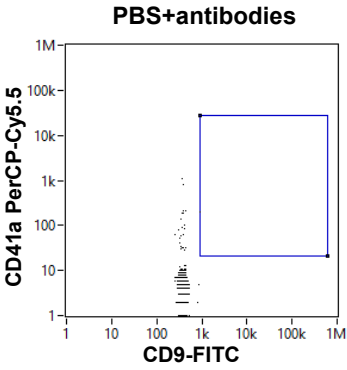

B

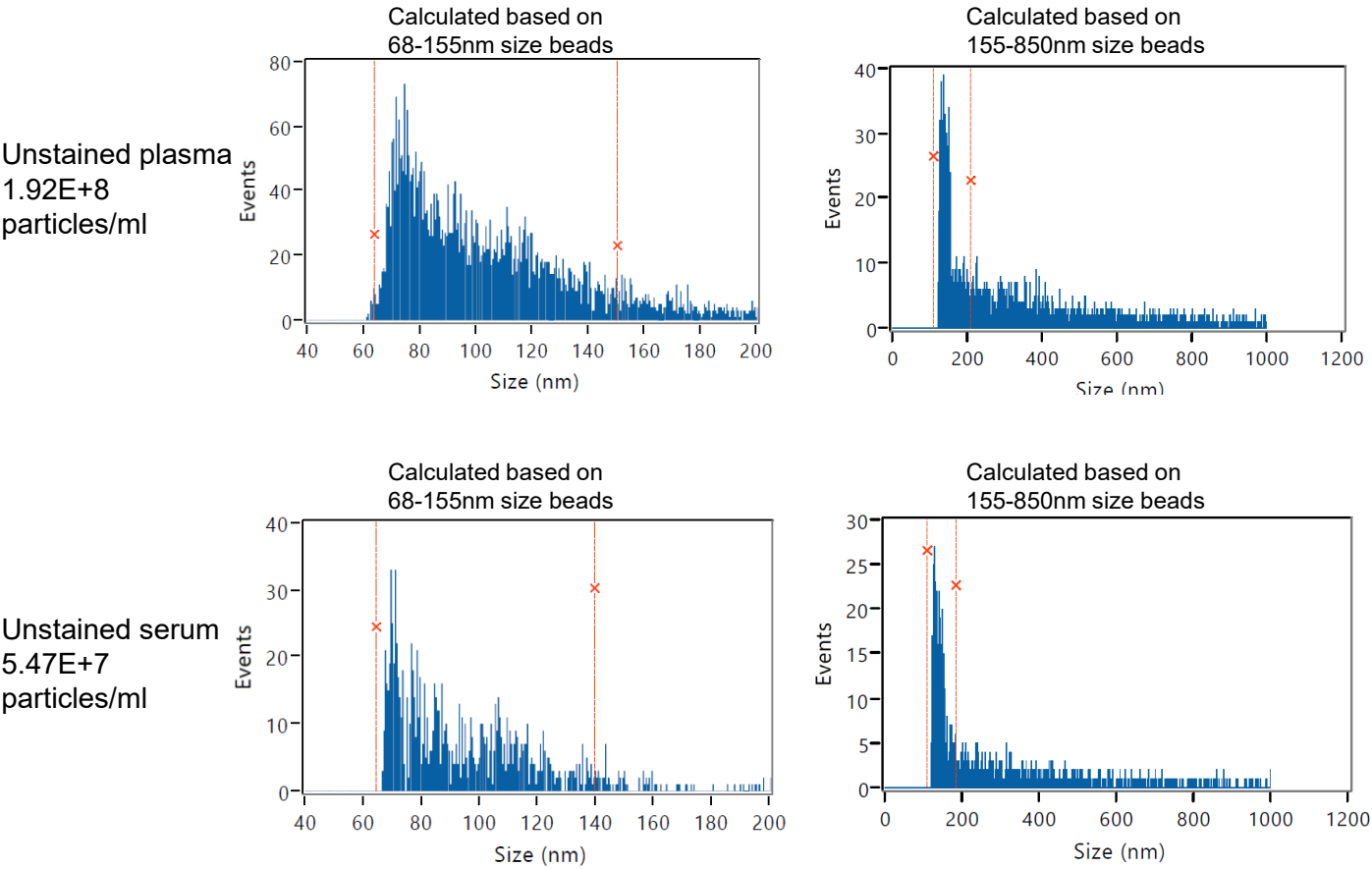

Supplement: Supplementary file 2 — SUPPORTING INFORMATION [file JEV2-11-e12213-s004.pdf]

# Supplementary Figure 3

A

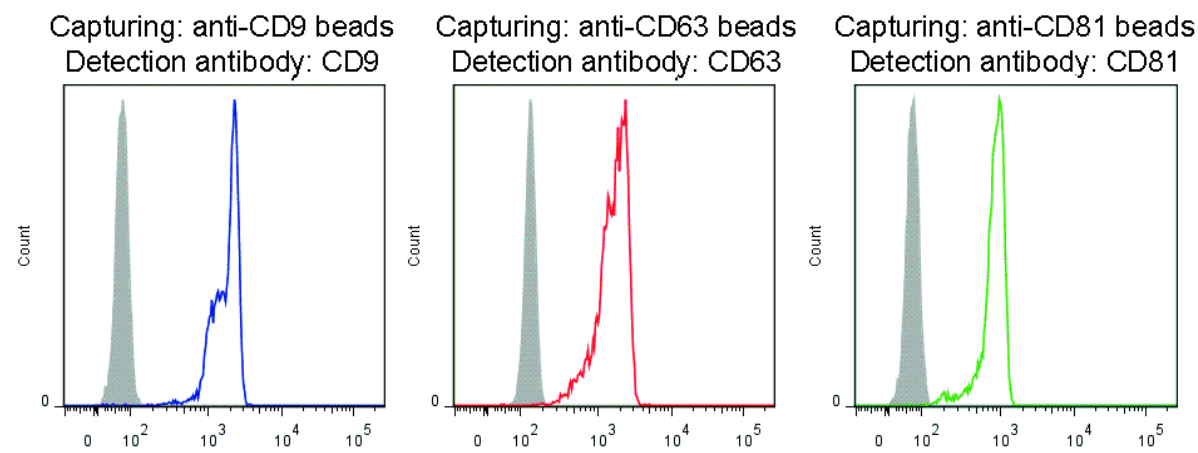

B

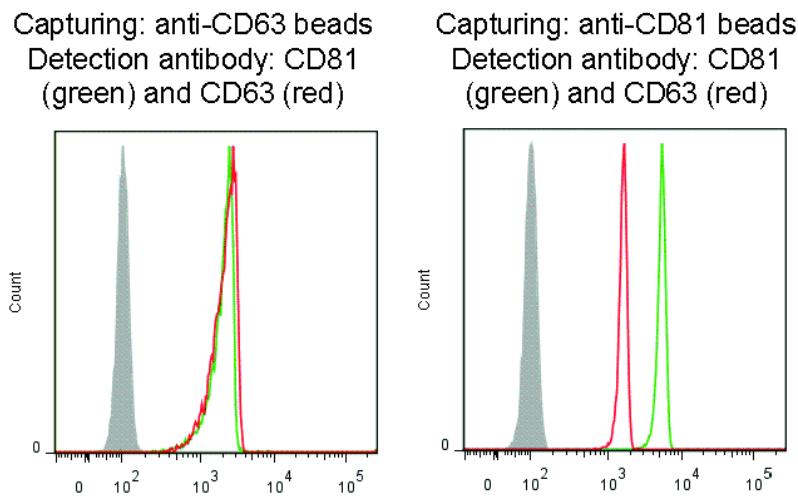

Supplement: Supplementary file 3 — SUPPORTING INFORMATION [file JEV2-11-e12213-s007.pdf]

# Supplementary Figure 4

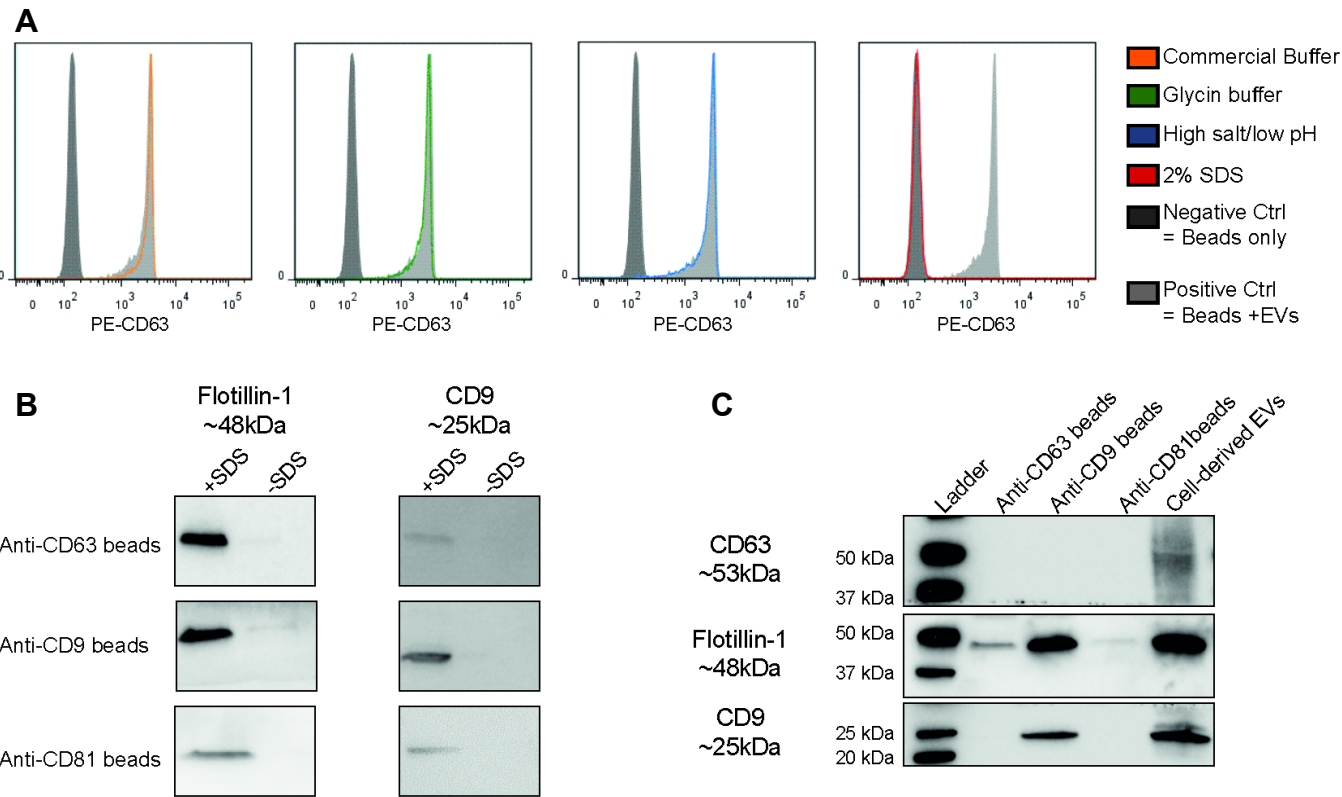

Supplement: Supplementary file 4 — SUPPORTING INFORMATION [file JEV2-11-e12213-s006.pdf]
